# Molecularly imprinted polymers (MIPs): emerging biomaterials for cancer theragnostic applications

**DOI:** 10.1186/s40824-023-00388-5

**Published:** 2023-05-13

**Authors:** Min Seok Kang, Euni Cho, Hye Eun Choi, Chaima Amri, Jin-Ho Lee, Ki Su Kim

**Affiliations:** 1 School of Chemical Engineering, Pusan National University, 2 Busandaehak-Ro 63 Beon-Gil, Geumjeong-Gu, Busan, 46241 Republic of Korea; 2 School of Biomedical Convergence Engineering, Pusan National University, 49 Busandaehak-Ro, Yangsan, 50612 Republic of Korea; 3 Department of Information Convergence Engineering, Pusan National University, 49 Busandaehak-Ro, Yangsan, 50612 Republic of Korea; 4 Department of Convergence Medical Sciences, School of Medicine, Pusan National University, 49 Busandaehak-Ro, Yangsan, 50612 Republic of Korea; 5 Department of Organic Material Science & Engineering, Pusan National University, 2 Busandaehak-Ro 63 Beon-Gil, Geumjeong-Gu, Busan, 46241 Republic of Korea; 6 Institute of Advanced Organic Materials, Pusan National University, 2 Busandaehak-Ro 63 Beon-Gil, Geumjeong-Gu, Busan, 46241 Republic of Korea

**Keywords:** Molecular imprinted polymer, Cancer, Theragnostic, Diagnostic, Drug delivery

## Abstract

Cancer is a disease caused by abnormal cell growth that spreads through other parts of the body and threatens life by destroying healthy tissues. Therefore, numerous techniques have been employed not only to diagnose and monitor the progress of cancer in a precise manner but also to develop appropriate therapeutic agents with enhanced efficacy and safety profiles. In this regard, molecularly imprinted polymers (MIPs), synthetic receptors that recognize targeted molecules with high affinity and selectivity, have been intensively investigated as one of the most attractive biomaterials for theragnostic approaches. This review describes diverse synthesis strategies to provide the rationale behind these synthetic antibodies and provides a selective overview of the recent progress in the in vitro and in vivo targeting of cancer biomarkers for diagnosis and therapeutic applications. Taken together, the topics discussed in this review provide concise guidelines for the development of novel MIP-based systems to diagnose cancer more precisely and promote successful treatment.

**Graphical Abstract:**

Molecularly imprinted polymers (MIPs), synthetic receptors that recognize targeted molecules with high affinity and selectivity, have been intensively investigated as one of the most attractive biomaterials for cancer theragnostic approaches. This review describes diverse synthesis strategies to provide the rationale behind these synthetic antibodies and provides a selective overview of the recent progress in the *in vitro* and *in vivo* targeting of cancer biomarkers for diagnosis and therapeutic applications. The topics discussed in this review aim to provide concise guidelines for the development of novel MIP-based systems to diagnose cancer more precisely and promote successful treatment.

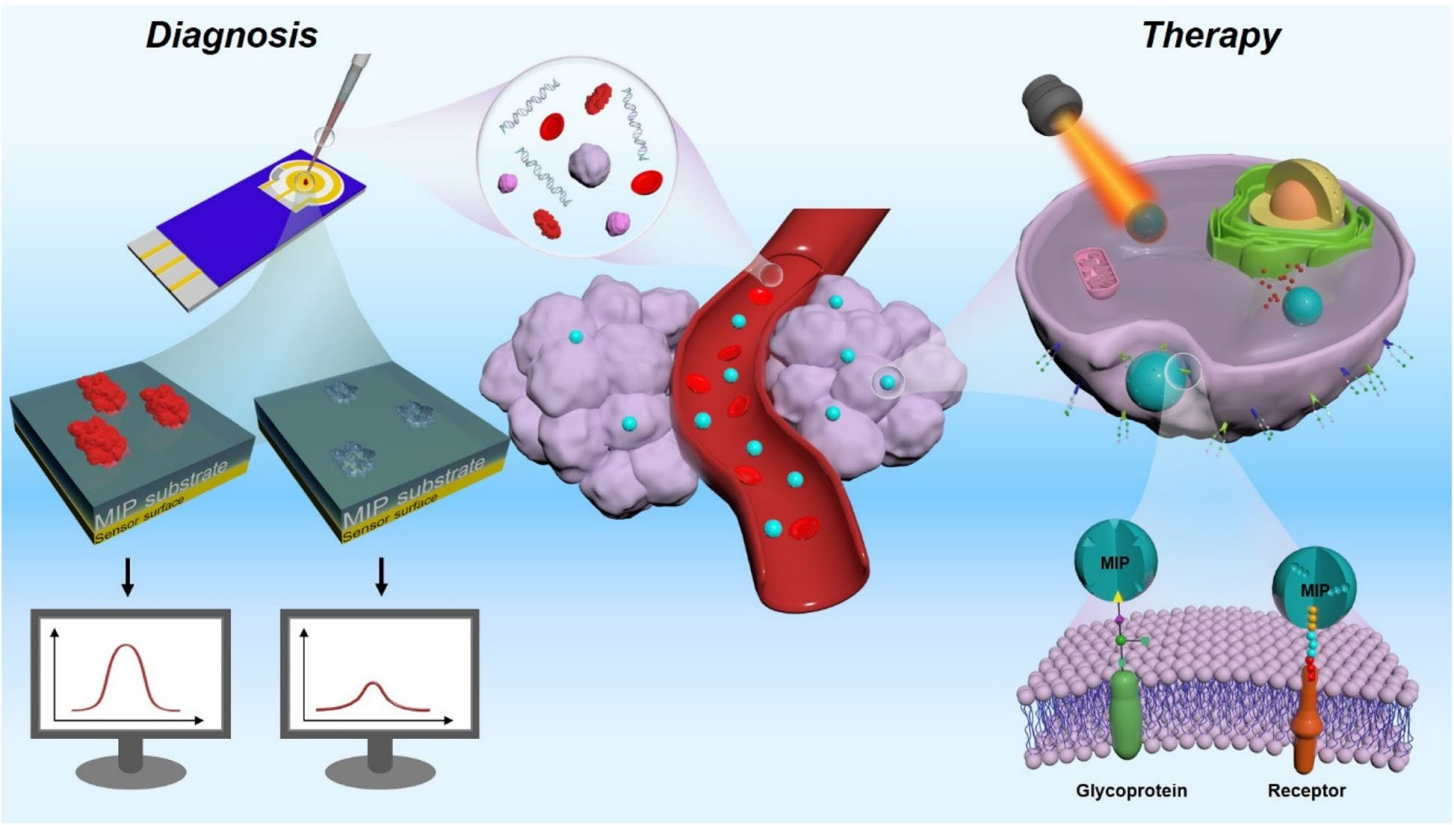

## Introduction

Cancer, one of the most severe diseases, starts from a malfunction at the single-cell level and proliferates throughout the body, destroying healthy tissue and eventually leading to death [1, 2]. More than often, the survival rate of a cancer patient highly depends on early diagnosis and efficient treatment [3–5]. Generally, the precise diagnosis of cancer relies on the histological evaluation of tissues, which can be laborious and time-consuming, thus making it difficult to diagnose cancer at an early stage [6, 7]. To this end, research in the oncologic field has shifted its interest toward the development of highly sensitive and selective biosensors to monitor cancer biomarkers in various biopsies [8, 9]. However, owing to their low concentration, especially in the early stages of cancer, highly sensitive and specific detection systems are needed for successful diagnosis and treatment [10, 11].

Among the emerging contenders, molecularly imprinted polymer (MIP)-based detection systems have shown promising results owing to their great adaptability, allowing for the more sensitive and selective detection of cancer biomarkers [12, 13]. Although traditional antibody and aptamer-based targeting materials present great specificity, they are produced through long and laborious selection processes, such as antibody screening or systematic evolution of ligands by exponential enrichment methods [14–16]. Whereas molecular imprinting of polymers only requires a high-purity sample of the target molecule and allows for the relatively straightforward synthesis of equally specific targeting materials at a lower cost [17, 18]. MIPs are based on the polymerization of a specific monomer and crosslinking agent in the presence of a template [19]. This molding process produces a specific structure that is selective to the molecule of interest used as a template. Furthermore, post-imprinting modifications in MIPs not only allow for custom tailoring of the polymer, but can also increase the affinity between the target and the MIP [20–22]. For instance, improved performance (i.e., sensitivity and detection range) of the ELISA (enzyme-linked immunosorbent assay) system was able to be achieved while utilizing MIP compare to antibodies specific to selective biomolecules [23]. Additionally, compared to antibodies or aptamers, these synthetic probes offer a greater advantage in terms of their stability, thus maintaining better structural integrity during storage [24]. Thus, this material can be adapted to a broader range of applications.

Although MIPs have had noticeable successes as detection probes and in the separation of complex biological samples, they have also been efficiently used in controlled release systems that can be harnessed in cancer therapy [25, 26]. By imprinting a target on the surface of carrier nanoparticles (NPs), the release of the cargo can be localized to the targeted body area, thereby reducing the toxic effect by using only the amount of drug required for therapeutic efficacies [27, 28]. Additionally, MIPs can be utilized for advanced therapeutic approaches by incorporating light-sensitive components [12, 29]. For example, heat can be generated by the exposure of a light-sensitive polymer to a certain light wavelength, leading to the localized thermal ablation of cancer tissue for photothermal therapy (PTT) [29, 30]. Moreover, MIPs exposed to photonic stimulation can locally produce cytotoxic reactive oxygen species (ROS) for photodynamic therapy (PDT) [31, 32]. Thus, owing to their target specificity and tailorable properties, MIPs have been extensively employed as a cancer therapy tool to improve the efficacy of the therapy and reduce the potential side effects of targeting healthy tissues [33].

**Fig. 1:**
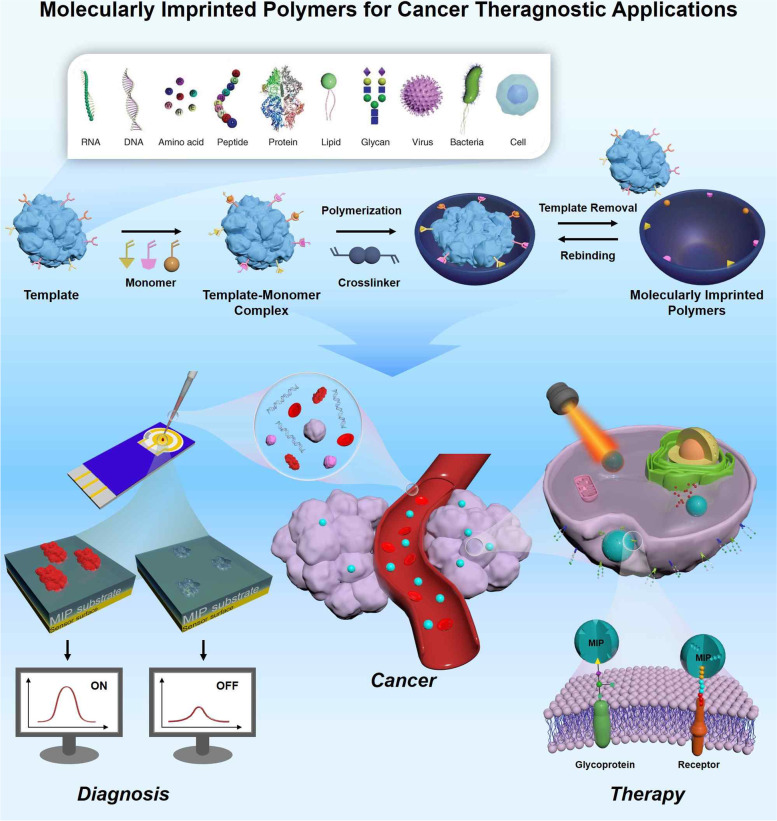
Schematic illustration of molecularly imprinted polymers for targeted cancer theragnostic applications

Although many reviews address cancer diagnosis and therapeutic approaches, as MIPs present an underrated profile for the development of more efficient cancer diagnostic methods and better-targeted therapy courses, they currently warrant a thorough review. This review provides an extensive examination of the current cutting-edge developments in the field (Fig. 1). Specifically, we will describe the different types of MIP production methods and the applications of the reported biosensors and therapeutic approaches, along with a critical discussion of the specific advantages and disadvantages. We believe that this article will pinpoint the most important challenges yet to be overcome and highlight research areas that require further investigation in the field. Moreover, by highlighting the most recent advancements, we hope that this article will incite interest from multiple disciplines and aid in providing a new perspective on the development of MIP-based advanced applications for cancer diagnosis and treatment.

## Strategies for the preparation of molecularly imprinted polymers

### Components utilized in molecular imprinted polymers

The ultimate goal of molecular imprinting is to create MIPs that can function similarly to biological receptors, allowing them to potentially replace these natural entities in certain applications. In order to create MIPs with a high degree of specificity and affinity to a particular target molecule, the molecular imprinting process involves the use of three essential reagents: template molecules, functional monomers, and cross-linkers. The selection of appropriate reagents plays a critical role in determining the level of affinity and specificity achieved in the final product.

#### Templates

The central importance of the MIP structure is a template that can direct organization of the functional group pendant to the selected functional monomers during molecular imprinting. In the performance of molecularly imprinted polymers, the presence of functional groups such as amino, carboxyl, hydroxyl, amide, and ester in the template is crucial [34, 35]. Generally, there are three requirements to satisfy the ideal template. First, the template molecule should exhibit excellent chemical stability during the polymerization reaction. Second, it should contain rich functional groups that can form template-monomer complex by combining with functional monomers. Finally, it is important to ensure that the binding between the template and monomer should not interfere with the polymerization or get destabilized during the polymerization reaction. So far, biological macromolecules such as proteins or cells were commonly used as templates in biomedical areas. This simple approach does not necessitate any complicated template preparation procedures and typically yields MIPs with suitable binding capacity and high selectivity [29, 36]. However, this approach for imprinting macromolecules has significant challenges, including the complexity of these molecules, the presence of non-specific recognition sites on their surfaces, limited polymerization techniques available for native macromolecules, and the potential for smaller non-target molecules to bind to the comparatively larger imprinted sites, resulting in reduced sensitivity [37–39]. To overcome these challenges, recent advancements have introduced the utilization of synthetic receptors, such as glycan, monosaccharide, oligosaccharide, epitope, or aptamer as templates for the molecular imprinting of target proteins.

#### Functional monomers

**Fig. 2:**
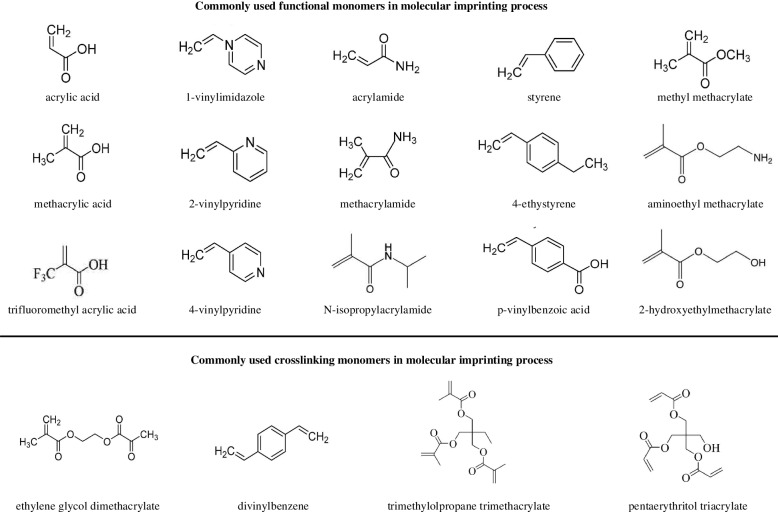
Structure of commonly used functional monomers and crosslinkers for molecular imprinting process

As shown in Fig. 2, due to the wide range of applications to MIPs, functional monomers and cross-linking agents to be used in MIP preparation are selected in consideration of the properties of the template (charge, size, chemical identity) [40]. The selection of appropriate polymers is one of the most important factors in molecular imprinting. Monomers typically contain two independent types of units: recognition units and units that can be polymerized. A key requirement for the selection of monomers is that they must have functional groups capable of interacting with the template. These groups can form covalent or non-covalent interactions, such as hydrogen bonding, dipole, van der Waals interaction, or π − π interaction, with the template to generate a template-monomer complex. The interaction between the template molecule and the functional group present in the polymer matrix induces molecular recognition [41, 42]. This process occurs in the pre-polymerization reaction, and it determines the quality and quantity of the recognition unit of a MIP [43]. Molecularly imprinted polymers are prepared by forming complexes when functional monomers react sufficiently with template molecules, followed adding crosslinking agents to immobilize functional groups of functional monomers on the imprinted molecules. A three-dimensional polymer network is then created only for the target template. So, the polymer must be sufficiently crosslinked to ensure that the binding site remains intact even after the template is removed [44].

#### Crosslinker

The incorporation of cross-linkers in the imprinting process enables the functional monomers to be closely arranged around the template molecules, giving rise to a polymeric matrix that is highly cross-linked. This enables accurate manipulation of the morphology and mechanical stability of MIPs after the templates are removed [43]. Similar to monomers, the type and number of cross-linker monomers used in the polymerization process also plays an important role in MIPs properties [45]. The mechanical stability and the number of recognition sites in MIPs are both dependent on the quantity of cross-linker utilized during the polymerization process; an insufficient amount of crosslinker can lead to unstable mechanical properties, while an excessive amount can decrease the number of recognition sites per unit mass of MIPs [35, 46]. Thus, it is necessary to optimize the ratio of basic cross-linkers beforehand to achieve the best polymerization results. In addition, the type of crosslinking agent determines the quality and yield of the MIP after polymerization. Proper combination and orientation of the monomers not only leads to improved affinity and high selectivity for the target template, but also determines the mechanical stability and porosity of the polymer.

### Imprinting strategies

Molecular imprinting consists of creating artificial recognition sites on polymeric matrices in which the size, shape, and spatial arrangement of the functional groups are complementary to the chosen templates, such as peptides, proteins, bacteria, and mammalian cells [36, 47]. In biological applications, MIPs can be used as tailor-made receptors for the recognition of biological substances such as antibodies and enzymes. Selecting the appropriate synthesis method is crucial in order to produce a MIP with required properties. To manufacture MIPs, there are different types of molecular imprinting techniques that can be classified in three main categories, including bulk imprinting, surface imprinting, and epitope imprinting.

#### Bulk imprinting

**Fig. 3:**
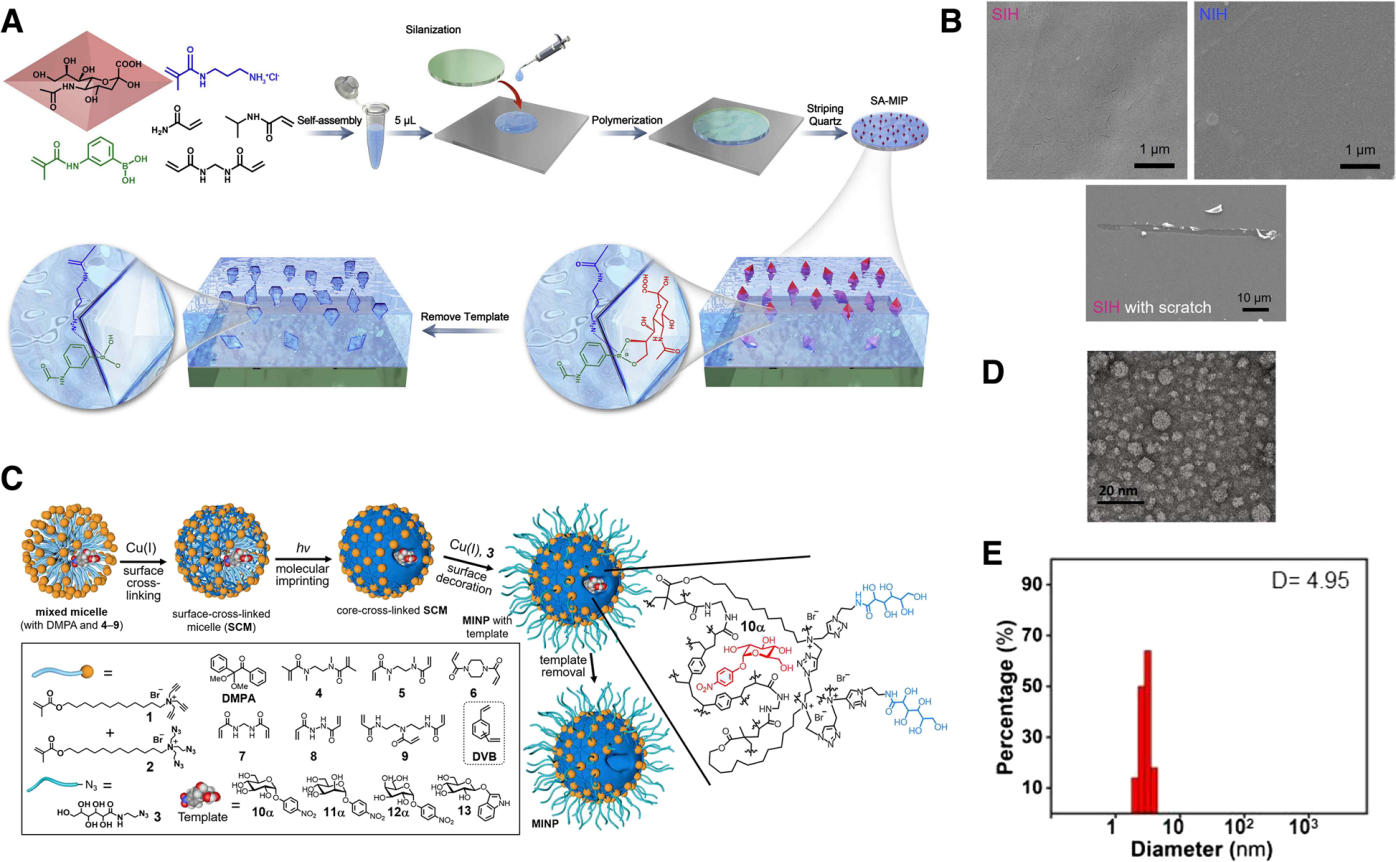
**A** Schematic illustration of procedure for preparing sialic acid (SA)-imprinted thermo-responsive hydrogel layer via bulk imprinting. **B** Scanning electron microscopy (SEM) images of the prepared imprinted hydrogel on quartz substrate and estimation of hydrogel layer thickness by a syringe needle. **C** Schematic illustration of the preparation of glycan-binding molecularly imprinted nanoparticles (MINPs) with crosslinked structure using a mixture of divinylbenzene (DVB) and MBAAm as the free radical cross-linker. **D** Transmission electron microscopy (TEM) image of the prepared MINPs. **E** Hydrodynamic size of MINPs in water determined by dynamic light scattering. **A**, **B** Reproduced with permission from [58], published by Elsevier 2021. **C**, **D**, **E** Reproduced with permission from [59], published by American Chemistry Society 2020

Bulk imprinting is the most well-known and straightforward method for molecular imprinting. In bulk imprinting, template molecules, functional monomers, initiators, and crosslinkers are mixed with solvents. After polymerization, all reagents are entirely converted into a solid polymer and the templates are removed. In the next step, to obtain the desired particle size and expose the recognition sites in the bulk material, post-treatment procedures, such as mechanical crushing, grinding, and sieving, are required [38]. This method produces high-purity imprinted materials through a quick and simple preparation process, without the need for complicated or costly instrumentation [29, 48]. So, the high purity of the product obtained through this method eliminates the need for additional purification steps, making it a sustainable and environmentally friendly approach. In this method, three-dimensional binding sites are formed for the entire template. This imprinting technique is particularly used in detection applications such as extraction [49–51], separation [52–54], and sensing [55–57]. Ma et al. synthesized a bulk imprinted thermo-responsive hydrogel layer via redox-initiated polymerization (ammonium persulfate [APS]/tetra-methylethylenediamine [TEMED]) at 37 ℃ in phosphate-buffered saline (PBS) using N-isopropylacrylamide (NIPAAm) as a thermo-responsive monomer, 3-methacrylamidophenylboronic acid as an affinity monomer, and N,N′-methylenebisacrylamide (MBAAm) as a crosslinker [58]. Polymerization occurred between a 3-(trimethoxysilyl)propyl methacrylate-functionalized quartz slide and a cover glass, resulting in the formation of a thin hydrogel layer on the quartz side. Poly(NIPAAm) served as the thermo-responsive hydrogel backbone for the selective capture and release of cancer cells (Fig. 3A, B). As an example of separation application used as a NP formulation, Zangiabadi et al. developed a convenient one-pot method to prepare molecularly imprinted micelle NP receptors to specifically distinguish between the subtle structural differences in glycans, glycosides, and even glycoproteins [59]. During synthesis, amphiphilic template molecules were spontaneously incorporated into the mixed micelles of cationic surfactants. Then, the mixed micelles were crosslinked on the surface owing to the numerous terminal alkynes and azides. This was followed by free radical polymerization (Fig. 3C-E). In this study, MIPs with strong hydrogen bonding interactions at the surfactant/water interface substantially enhanced the binding of molecularly imprinted NPs (MINPs) to afford micromolar affinities for complex glycans and glycoproteins equivalent to those achieved by natural lectins. In bulk imprinting, however, post-treatment steps that have to obtain small particles and create a large surface area for recapture are time consuming and result in non-uniform sizes and shapes, as well as heterogeneities in the binding sites repartition [60]. Additionally, in the case of large molecules, including macromolecules, proteins, living cells, and microorganisms, their diffusion to molecular cavities buried inside an MIP matrix is hindered significantly [61]. This hindrance results in the low sensitivity and selectivity of MIP-based bio-applications. Hence, alternative imprinting techniques have been developed to overcome these drawbacks.

#### Surface imprinting

**Fig. 4:**
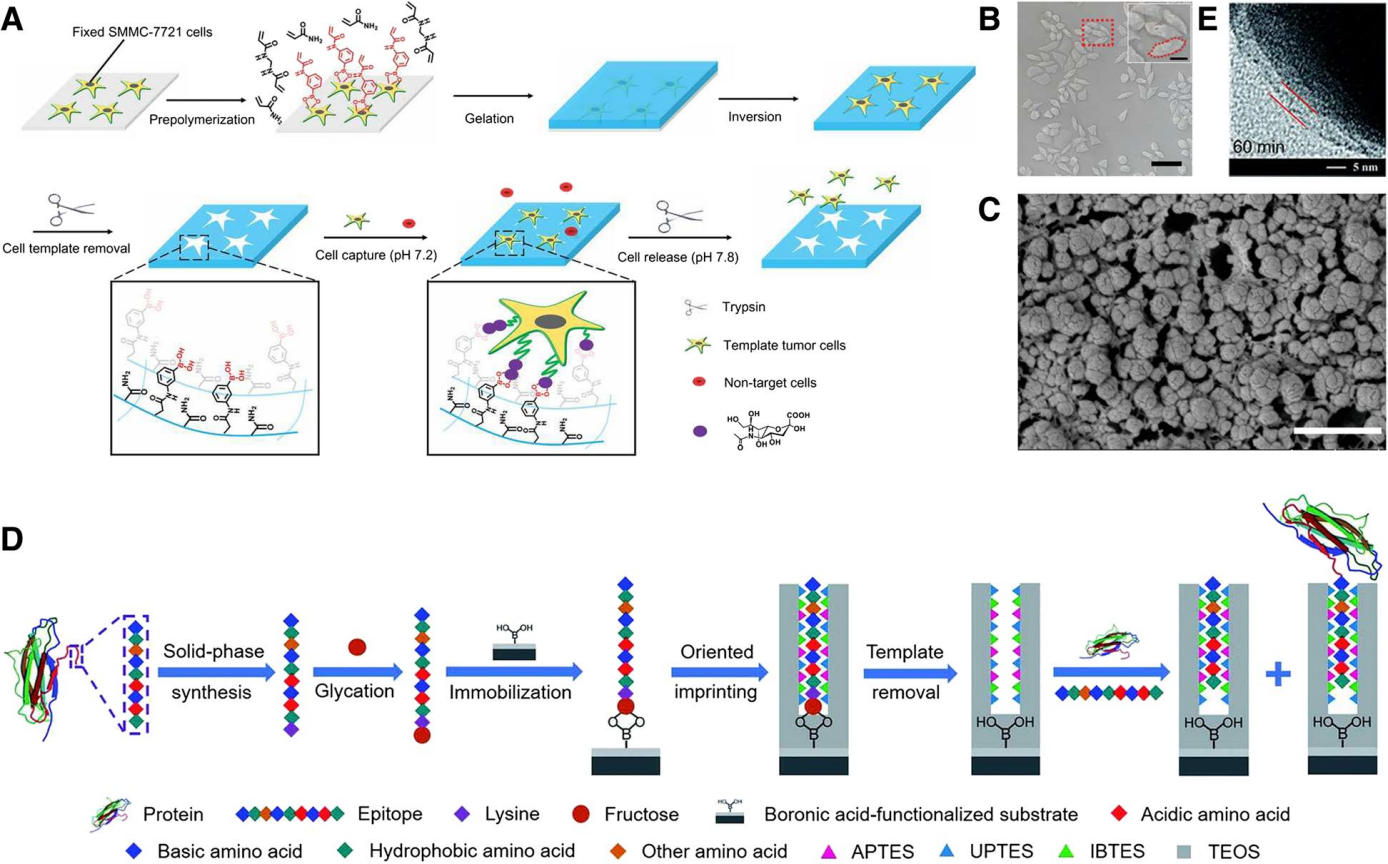
**A** Fabrication of the hydrogel with boronate affinity via imprinting of SMMC-7721 cells for the capture and release of tumor cells. **B** Microscopy images of surface morphology of the imprinted hydrogel with fixed cells. **C** Cryo-scanning electron microscopy (SEM) of the nanostructures inside the imprinted hydrogel. **D** Schematic of the principle and procedure of controllable oriented surface imprinting of boronate affinity-anchored epitopes. **E** Transmission electron microscopy (TEM) image of the imprinting layer of silver NPs (AgNPs) imprinted with 60 min polymerization time. **A**, **B**, **C** Reproduced with permission from [71], published by Wiley 2020. **D**, **E** Reproduced with permission from [77], published by The Royal Society of Chemistry 2019

In surface imprinting, the template is located at the surface or in the proximity of the polymeric framework’s surface to ensure more effective recognition [38]. Compared with bulk imprinting (Table 1), it can provide high accessibility of the template molecules to the binding sites of MIPs, resulting in rapid recognition. In addition, the diffusion distance of the target to the binding site and the steric hindrance of the molecules are minimized [12], which is especially beneficial in (bio)macromolecule imprinting. In summary, surface-imprinted nanomaterials show an extremely high surface-to-volume ratio and rapid template transfer, consequently improving the binding capacity and kinetics. Moreover, it can provide additional features by integrating other functions. Therefore, synthesized MIPs have been used in a wide range of applications [62], including affinity separation [63–65], sensing [66–68], and drug delivery systems [27, 69]. Surface imprinting can be divided into two steps: (i) immobilization of the protein template on the solid substrate surface and (ii) polymerization of a thin polymer layer around the immobilized template molecules [70]. To generate an imprinting layer on a surface, functionalization steps are necessary for protein immobilization. For example, boronic acid functionalization for oriented glycoprotein immobilization and iminodiacetic acid (IDA)-Cu^2+^ introduction to immobilize surface His-containing proteins are two well-established protocols [12, 62]. Subsequently, a thin layer of polymer was formed to cover the templates, followed by the elimination of template molecules by washing with solvents. Liu et al. proposed a universal strategy for the fabrication of an antibody-free biomimetic hydrogel via cell imprinting (Fig. 4A-C) [71]. The polymerized functional monomer, crosslinker, and affinity monomer (3-[acrylamido]phenylboronic acid [3-AAPBA]) were added to the culture dish in the presence of adhered human hepatocarcinoma SMMC-7721 cells to form a hydrogel. By integrating 3-AAPBA into the cell imprinting process, the synergistic effect of cell imprinting and boronate affinity was realized in one step. After peeling off the hydrogel and removing the cells, the imprinted surface selectively captured and released undamaged tumor cells. Although surface imprinting addresses the problems encountered in macromolecular mass transfer, whole proteins are still difficult to use efficiently for imprinting owing to their complex structure, numerous functionalities, and risk of denaturation [62].

**Table 1:** Comparison of bulk imprinting and surface imprinting

Method	Bulk imprinting	Surface imprinting	Reference
**Fabrication accessibility**	Easy	Complicated	[36, 72]
**Template conformational maintence**	Usually hard	Easy	[46]
**Template utilization efficiency**	Usually low	Usually high	[38, 73]
**Template removal**	Hard	Complete removal	[74, 75]
**Rebinding capacity**	Low	High	[36, 76]
**Recognition site accessibility**	Low	Easy	[35, 39]

Epitope surface imprinting technology aims to prepare imprinted materials by controlling the epitope template such that it is located at the material surface [48]. An epitope is a distinctive sequence of amino acids within the structures of globular proteins that reflects their structural specificity [78]. Thus, this method can produce MIPs with templates containing protein epitopes, decreasing the complexity of the protein structure. Epitope-imprinted polymers can distinguish epitope peptides through specific sequential or conformational epitopes, as well as recognize whole proteins in real samples. If the right epitope is selected, epitope imprinting has various advantages, including an abundant choice of templates, improved applicability under a variety of reaction conditions, and a defined orientation to bind to the target proteins [79]. Xing et al. introduced a novel strategy based on controllable oriented surface imprinting, utilizing a glycated C-terminus nonapeptide epitope as a template, which could facilitate the immobilization and removal of templates on the surface of a boronic acid-functionalized substrate [77]. C-terminus nonapeptides for human β_2_-microglobulin (B2M) and myoglobin, attached with a lysine, were used as target proteins. Epitope imprinting was conducted via a polycondensation reaction using multiple silylating reagents containing functionalities interacting with the epitope, including aminopropyltriethoxysilane (APTES), 3-ureidopropyl-triethoxysilane, isobutyltriethoxysilane, and tetraethyl orthosilicate (TEOS) (Fig. 4D, E). The resulting MIPs exhibited a strong affinity and high specificity toward B2M. Guo et al. developed coreless core/shell NPs with a specific targeting capability toward proteins and peptides via a novel approach called reverse microemulsion-confined epitope-oriented surface imprinting and cladding [80]. N- or C-terminal nonapeptides grafted with a hydrophobic fatty acid chain were used as templates. In the process of reverse microemulsion formation, the epitope is anchored at the microemulsion interface, and then silylating monomers are added to the aqueous phase and polymerized. After polymerization, TEOS is added to the microemulsion mixture to form a thin hydrophilic silica cladding layer. The resulting MIPs successfully differentiated cancer cells from other cell lines. Although surface imprinting and epitope imprinting have addressed the issues of adsorption efficiency and protein targeting, there are still several possibilities for improving the solvent compatibility, template removal, and material recyclability.

Since the molecular imprinting layer can sufficiently maintain morphological integrity and can be reused, many studies are being conducted to be used in sensing platforms. In addition, it can be used as a controllable drug delivery platform through smart polymers that cause morphological deformation in response to stimuli [81, 82]. MIP materials can be selected according to the situation, and the thickness of the imaging layer can be adjusted by adjusting the reaction time to generate the appropriate thickness required for sensing and treatment [83]. MIPs possess superior physical strength, heat resistance, and pressure resistance, as well as greater resistance to harsh environments, when compared to biological systems like proteins and nucleic acids [84]. Moreover, the cost of synthesis is inexpensive, they can be effortlessly tailored to meet specific needs, and the recognition capacity can be preserved for numerous years at room temperature, contributing to an extended shelf life for the polymers [85]. As described in the following chapter, the unique properties of MIP materials are beneficial to develop various cancer theragnostic applications.

## MIP-based cancer diagnosis

Tissue biopsy is currently the most accurate and definitive method for cancer diagnosis [86]. However, such an invasive method not only can be costly, but also provides an analysis limited to a specific area of sample collection [87–89]. Furthermore, repeated interventions are not feasible; thus, accurate monitoring of cancer evolution is not possible [90]. The detection of circulating cancer biomarkers through liquid biopsies offers a low-cost, simple, and non-invasive alternative to this problem [6, 87, 91, 92]. In vivo imaging of cancer biomarkers is another possible alternative to tissue biopsy [93]. Either way, MIP-based sensors and imaging probes have demonstrated great results for the detection of cancer biomarkers, such as nucleic acids, proteins, exosomes, and cancer cells [94–96]. As each biomarker comes with its own detection challenges, MIP-based technologies, as well as offering great stability and sensitivity, present an unprecedented adaptability of the detection probes [96–98].

### Nucleic acid

**Fig. 5:**
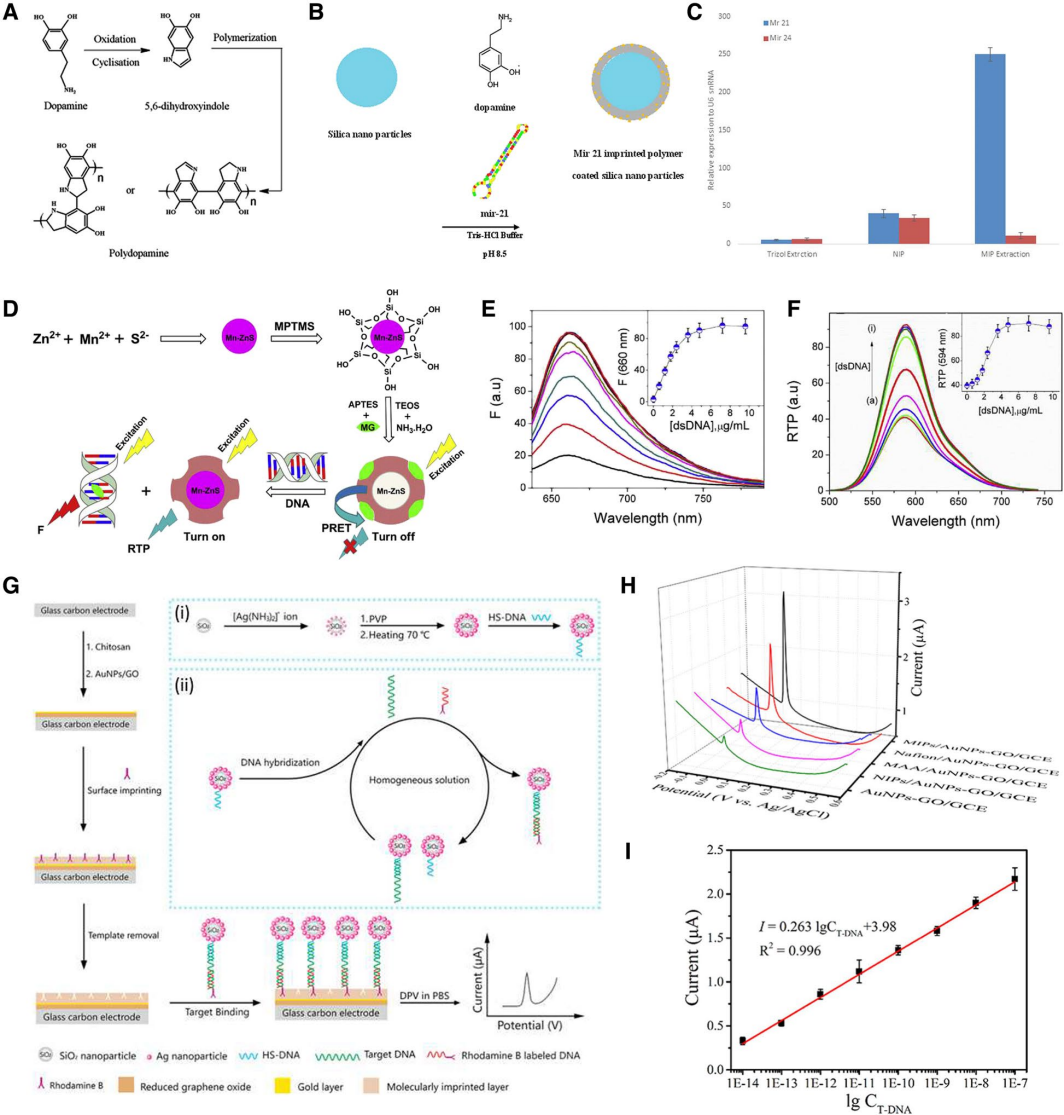
**A** Reaction mechanism of polymerization of dopamine. **B** Preparation of mir-21 imprinted polymer coated silica nanoparticles. **C** miR-21 relative expression in the U-87 MG cell lysate using, Trizol reagent, NIP and MIP. **D** Synthesis of MIP-quantum dots (QDs), and their interactions with double-stranded DNA (dsDNA). **E** Fluorescence spectra of MG (7.0 μM) in the presence of MIP-QDs (0.1 mg/mL) upon addition of dsDNA in buffer (pH 7.4). Inset: Change in fluorescence emission intensity of MG at 660 nm depending on dsDNA concentration. **F** RTP spectra of MIP-QDs in the presence of MG as a function of dsDNA concentrations; a) 0 ~ i) 9.6 μg/mL. Inset: Change in RTP intensity of MIP-QDs at 594 nm with dsDNA addition. **G** Schematic illustration of the MIPs-based E-DNA biosensing. Inset of (i) and (ii) display the preparation of SiO2@Ag/DNA and homogeneous DNA hybridization. **H** DPV responses of AuNPs-GO/GCE, NIPs/AuNPs-GO/GCE, MAA/AuNPs-GO/GCE, Nafion/AuNPs-GO/GCE, and MIPs/AuNPs-GO/GCE in 0.1 mM PBS (pH 7.4) after incubation with SiO2@Ag/dsDNA/RhoB for 35 min. **I** calibration curve used for detection of T-DNA from 10 fM to 100 nM. **A**, **B**, **C** Reproduced with permission from [101], published by Elsevier 2020. **D**, **E**, **F** Reproduced with permission from [102], published by John Wiley and Sons 2016. **G**, **H**, **I** Reproduced with permission from [103], published by Elsevier 2018

Nucleic acid, deoxyribonucleic acid (DNA), and ribonucleic acid (RNA) have long been used as markers for cancer diagnosis because they are often released into the bloodstream after cell apoptosis [99, 100]. Hashemi-Moghaddam et al. focused their work on the separation of microRNA 21 from glioblastoma cell line lysates (Fig. 5A-C) [101]. A layer of dopamine was polymerized on the surface of the silica NPs to synthesize capture probes. Then, the outcome of the miR-21 extraction comparison among MIP, NIP, and Trizol reagents confirmed that the synthesized polymer selectively entrapped miR-21, leading to an upregulation of miR-21 expression in the final eluate.

It has been established that the majority of DNA associated with tumor exosomes is double-stranded (dsDNA). Thus, dsDNA detection can serve as a cancer diagnosis and preoperative assessment tool. Arslan et al. developed a radiometric sensor for the fluorescent detection of dsDNA (Fig. 5D-F) [102]. Mercapto-propyl-trimethoxy-silane-capped Mn-doped ZnS quantum dots (QDs) were synthesized and imprinted through sol–gel polymerization. This imprinting method relies on the gelation of a colloidal suspension to form a polymeric network around the template of interest, in this case a cationic dye, malachite green (MG). The sensing mechanism consisted of a “turning off” mode where the fluorophore’s room temperature phosphorescence (RTP) was quenched through phosphorescence resonance energy transfer, and a ‘turned on’ mode where the presence of dsDNA would induce the release of MG molecules and an increase in the RTP signal proportional to the target availability. Through their method, a limit of detection (LOD) of 19.48 ng/mL was achieved under optimized experimental conditions. Furthermore, spiked urine samples were successfully tested to validate the applicability of the sensor to real samples.

As a similar but different approach, what MIP actually catches is an antibody, but then there is a method of detecting the target gene through the antibody. You et al. designed an MIP-based electrochemical biosensor for the detection of *BRCA-1*, the gene encoding for breast cancer type 1 susceptibility protein, in serum samples (Fig. 5G-I) [103]. The sensing platform was built on a glass carbon electrode (GCE) modified with gold NP-reduced graphene oxide. An MIP film coated the surface of the GCE with rhodamine B (RhoB) as a template and a mixture of methacrylic acid (MAA) and Nafion as the monomer and additive, respectively. By choosing this polymer composition, the team aimed to improve the electrostatic interaction between rhodamine B and MIPs. The second element of the detection mechanism consisted of fabricating a signal amplification tracing tag. The synthesized monodisperse silica NPs were covered with silver NPs (AgNPs). This step was followed by the binding of the DNA probe to the surface of the AgNPs. In the presence of the target DNA, homogenous hybridization would occur between the silica NP probe and an RhoB-labeled nucleic acid fragment. The electrochemical detection of the hybridized probe was performed through differential pulse voltammetry (DPV) and demonstrated excellent selectivity, reproducibility, and stability with a low detection limit of 2.53 fM. Despite the interesting approaches taken for the detection of nucleic acids as cancer biomarkers, there are a lack of studies on the direct quantification of this category of biomarker in biological samples.

### Protein

**Fig. 6:**
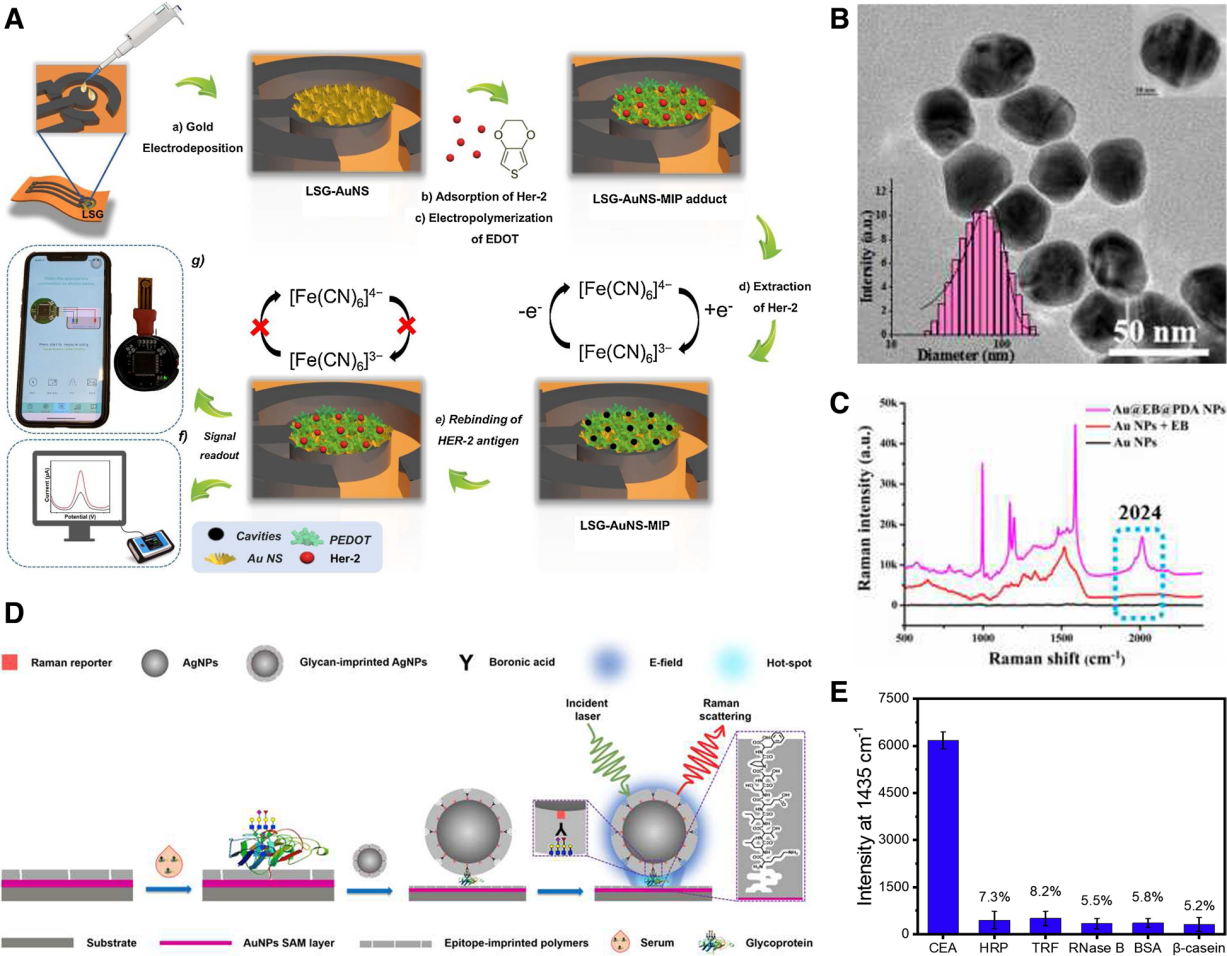
**A** Schematic illustration of the Laser scribed graphene (LSG)-AuNS-MIP based biomimetic sensor for Her-2 detection. **B** TEM image of Au@PDA NPs. Inset: hydrodynamic diameters of Au@EB@PDA NPs. **C** Optimization of Au@EB@PDA NPs with Raman signal at a silent zone at 2024 cm − 1. **D** Schematic illustration of orthogonal dual molecularly imprinted polymer-based plasmonic immunosandwich assay (odMIP-PISA) approach for detection of glycoprotein. **E** Compared specificity of proposed odMIP-PISA method for target CEA to various proteins. **A** Reproduced with permission from [104], published by Elsevier 2021. **B**, **C** Reproduced with permission from [105], published by Elsevier 2019. **D**, **E** Reproduced with permission from [106], published by Elsevier 2019

Protein detection probes are among the most explored applications for MIPs. These extremely dynamic molecules have been successfully used as cancer biomarkers owing to their high bioavailability, with a great number of studies demonstrating their potential as cancer diagnosis, prognosis, and treatment monitoring tools. Lahcen et al. focused on the detection of human epidermal growth factor receptor 2 (HER2), a breast cancer marker protein (Fig. 6A) [104]. The team developed a gold nanostructure-modified MIP-based laser-scribed graphene sensing platform with an LOD of 0.43 ng/mL. To fabricate the electrode, a polyimide sheet was exposed to a CO_2_ laser, and after the isolation of the areas of interest, a gold layer was formed through electrochemical deposition. In the synthesis of the MIP layer, HER2 was left to adsorb onto the platform before being covered with 3,4-ethylenedioxythiophene. Electro-polymerization of the film was performed through chronoamperometry, followed by the removal of the template molecule. The biosensor offered high sensitivity and selectivity for HER2 and was further integrated with an electrochemical analyzer.

Owing to the high sensitivity, low cost, and simplicity of electrochemical biosensors, numerous researchers have turned their attention to MIP-based electrochemical sensing. Lai et al. developed an electrochemical sensor for the detection of carcinomaembryonic antigen (CEA) [97]. Polydopamine (PDA) was used as the polymer through multiple electro-polymerization cycles to form the CEA template. The binding properties of the MIP were tested through DPV. For electrochemical detection, a glass carbon electrode was coated with polythionine and gold NPs (AuNPs). Lai et al. achieved an even lower LOD of 0.2589 pg/mL [97]. However, Lin et al.’s detection method had a greater success with an LOD of 0.064 pg/mL for CEA in real blood samples (Fig. 6B, C) [105]. To achieve these results, the team combined a surface-enhanced Raman spectroscopy substrate with a surface-molecularly imprinted polymer (SMIP) target capture method. By encapsulating ethynylbenzene (EB) into dopamine (DA) on the surface of AuNPs and combining them with an imprinted core-molecule-shell-molecule NP-coated surface, the internal standard of the biosensor was optimized with a silent zone at 2024 cm^−1^. Zhou et al. took a slightly different approach to CEA detection by combining two types of MIPs in an immuno-sandwich assay (Fig. 6D, E) [106]. One MIP was coated on the gold NP layer designed to recognize the peptide epitope, whereas the second MIP covered the Raman-active silver NPs to capture the protein itself. This orthogonal double recognition method seemed to improve the specificity of the sensing mechanism, as it was tested on multiple proteins such as horseradish peroxidase (HRP), human apo-transferrin, ribonuclease B (glycoproteins), bovine serum albumin, and β-casein (non-glycoproteins) [106].

Prostate cancer can be detected by measuring prostate-specific antigen (PSA) levels in the blood of patients. Wang et al. developed a ‘signal-off’ electrochemiluminescence sensor for the detection of PSA at concentrations as low as 3.0 pg/mL [107]. In their experiment, oligonucleotide was self-assembled on the AuNPs functionalized glass carbon electrode. This was followed by the electropolymerization of DA hydrochloride to form MIP layer. The sensing probe achieved a detection limit of 3.0 pg/mL [107]. However, as it is still difficult to establish an accurate diagnosis simply based on the overexpression of PSA, Karami et al. designed a gold screen-printed electrode to simultaneously detect PSA and myoglobin, a biomarker often co-expressed with PSA, in human serum and urine samples of prostate cancer patients [108]. Cancer antigen 125 (CA-125), a glycoprotein of the mucin family, is an important biomarker for ovarian cancer. Rebelo et al. engineered an MIP-based screen-printed electrode for the detection of CA-125 [109]. Similar to previously reported methods, a monomer, in this case pyrrole, was electropolymerized on a gold electrode through cyclic voltammetry with CA-125 as a protein template. Interestingly, both surface plasmon resonance and electrochemical detection methods were evaluated, with the latter method achieving the lowest LOD at 0.01 U/mL [109]. Overall, proteins are excellent templates for the synthesis of membrane antibodies in cancer detection systems; they have multiple functional sites that can be easily reproduced. However, one of the most challenging aspects of these biomarkers is to maintain their native conformation during the imprinting process. Overcoming this problem could significantly improve the binding selectivity of MIPs.

### Exosomes

**Fig. 7:**
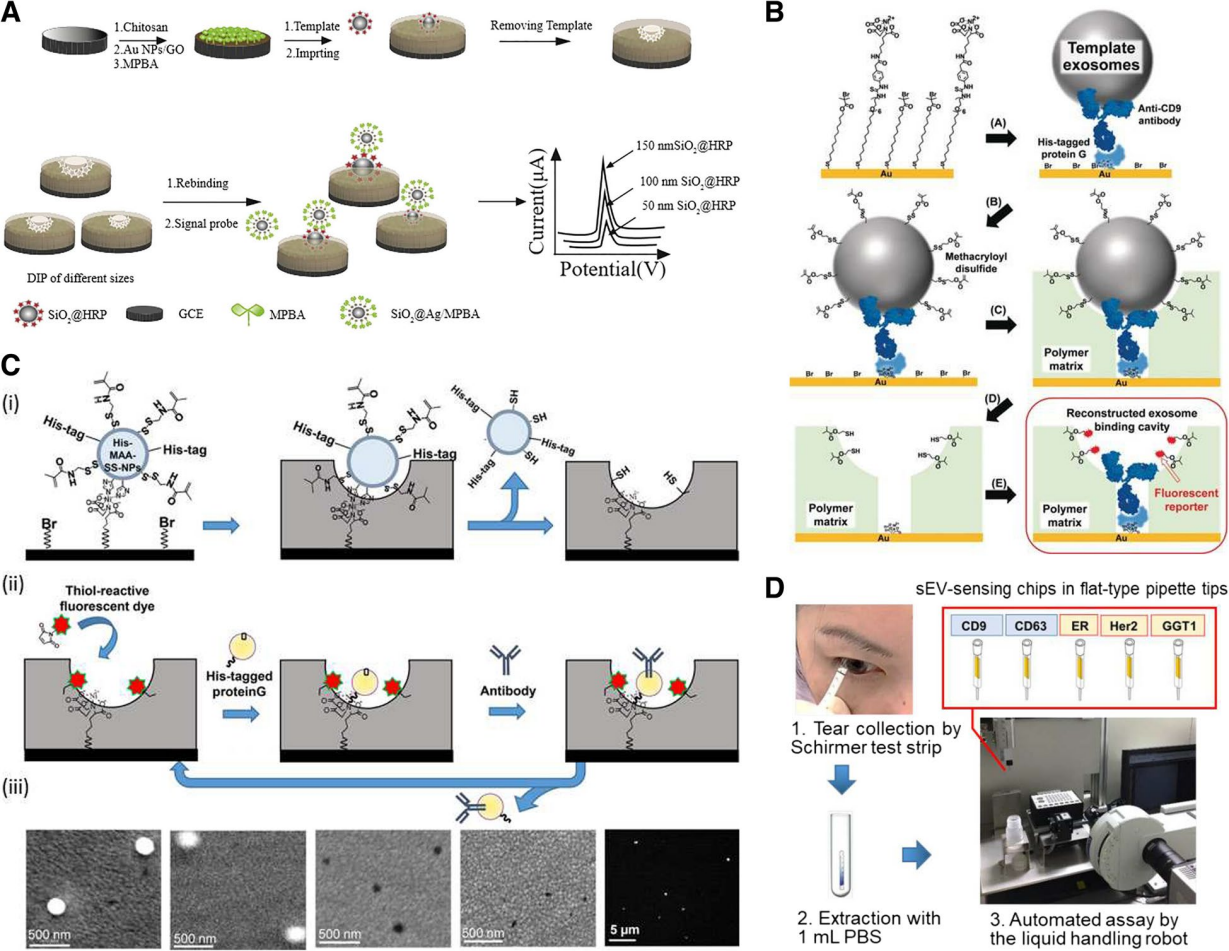
**A** Schematic illustration of double imprinting-based electrochemical detection of mimetic exosomes with size distributed nanoparticles. **B** Preparation of the exosome-binding cavity by molecular imprinting with post-imprinting in-cavity modifications (PIMs). **C** Antibody-conjugated signaling nanocavities for sensing intact small extracellular vesicles (sEVs) fabricated by molecular imprinting-based dynamic molding chemical nanoprocessing. (i) Dynamic molding nanoprocessing. (ii) Postchemical nanoprocessing. (iii) Scanning electron microscopic and fluorescence images during the dynamic molding nanoprocessing approach. **D** Tear sEV detection procedure. **A** Reproduced with permission from [110], published by Elsevier 2020. **B** Reproduced with permission from [112], published by John Wiley and Sons 2019. **C**, **D** Reproduced with permission from [113], published by The American Chemical Society 2020

When it comes to targets such as exosomes, the challenge resides in the irregularity of template size and shape. More than often, dull templates are used as substitutes for extracellular vesicles. This is because of the difficulty in extracting a homogeneous sample of exosomes for use as a template. For instance, Zhu et al. developed a sensor based on the double imprinting polymer method for the construction of an electrochemical detection platform for the particle size distribution of exosomes (Fig. 7A) [110]. AuNP-graphene oxide-modified glassy carbon electrodes were coated with a layer of 4-mercaptophenylboronic acid. To achieve double imprinting, HRP-coated silica NPs of different diameters (50, 100, and 150 nm) have served as templates. Despite the lack of application of the sensing mechanism to biological samples, the developed detection mechanism allowed for successful and reproducible analysis of mimetic exosome size ratio. With the technology of Liu et al., exosomes from cell culture media and urine samples can be efficiently isolated [111]. Owing to the difficulty faced in purifying exosomes for use as a mold, the capture mechanism was based on dull template imprinting with similar proprieties to the extracellular vesicles. Negatively charged silica NPs with a size distribution between 40 and 160 nm were effectively molded into a mixture of 2-(diethylamino)ethyl acrylate, and acrylamide monomers were combined with N,N′-methylene diacrylamide, a hydrophilic cross-linker. Once the template is removed, through the combination of both conformational recognition and electrostatic interaction, the artificial antibody is comparable to commercially available precipitation methods for exosome capture [111].

Another way to overcome the surface irregularities of exosomes is to combine the usage of MIPs with specific antibodies and aptamers. Mori et al. designed a molecularly imprinted sensing platform for the detection of prostate cancer-derived exosomes (Fig. 7B) [112]. A His-tagged protein G was used to bind CD9 targeting antibody to the gold surface of the sensor. It should be noted that CD9 is a protein abundantly expressed on the surface of exosomes. Template exosomes secreted from a prostate cancer cell line, PC3, were immobilized on the surface. This step was followed by the anchoring of methacryloyl disulfide groups onto the vesicles to allow for post-imprinting modifications after polymerization of the surrounding matrix and the removal of both exosomes and antibodies. Takeuchi et al. used a molecular imprinting-based dynamic molding technique to fabricate antibody-conjugated signaling nanocavities for the detection of exosomes secreted in the tears of breast cancer patients with an analysis time of 5 min per sample (Fig. 7C, D) [113]. In their experiment, they used a mold based on methacrylamide-coupled His-tagged silica NPs attached to the substrate through a Ni(II)-nitrilotriacetic acid bond. To minimize non-specific binding, a 20-nm thick polymer matrix was formed through the surface-initiated atom transfer radical polymerization of 2-methacryloyloxyethyl phosphorylcholine. During the post-chemical processing of the cavity, a fluorescent reporter molecule was added through a thiol bond, followed by the addition of a His-tagged protein G that allows for the insertion of the desired antibody.

The application of MIPs to pre-existing sensing mechanisms can significantly improve their sensitivity. Feng et al. developed a fluorescence resonance energy transfer system (FRET) for the detection of exosomes in the serum of breast cancer patients with an LOD of 2.43 × 10^6^ particles/mL [95]. The sensor consisted of two elements that formed a sandwich in the presence of the target of interest: an MIP capture element and an aptamer/graphene oxide (GO) selective ‘turn-on’ component. Fe_3_O_4_ NPs were used as a base for molding the extracellular vesicles within a mixture of APS and TEMED. The GO quenched the fluorescence of the aptamer-bound FAM; however, in the presence of the target, the oligonucleotide would be released from the GO and the fluorescence would increase proportionally to the availability of the analyte. Liao et al. developed the ‘turn-on’ fluorescent sensor to detect the presence of exosomes in patient serum with a sensitivity comparable to the enzyme-linked immunosorbent assay [94]. The detection process was achieved through aptamer-mediated aggregation-induced emission. The crosslinking of TEMED and APS via a cross-linking agent on the surface of magnetic microparticles allowed the molding of the target exosomes. After exposure to patient-derived serum, the beads were magnetically collected and then exposed to the mixture of aptamer and fluorogen (TABD-Py) to mediate aggregation induced emission. This sensing method achieved a detection limit of 1.3 × 10^6^ particles/mL. Owing to the variable sizes, shapes, and surface proteins of exosomes, the development of highly effective MIPs will most likely require more research on post-polymerization surface modifications. Nonetheless, the detection and capture of these vesicles and analysis of their proteomes can provide great insight into cancer diagnostics and prognostics, thus highlighting the importance of developing better MIP fabrication methods.

### Cells

**Fig. 8:**
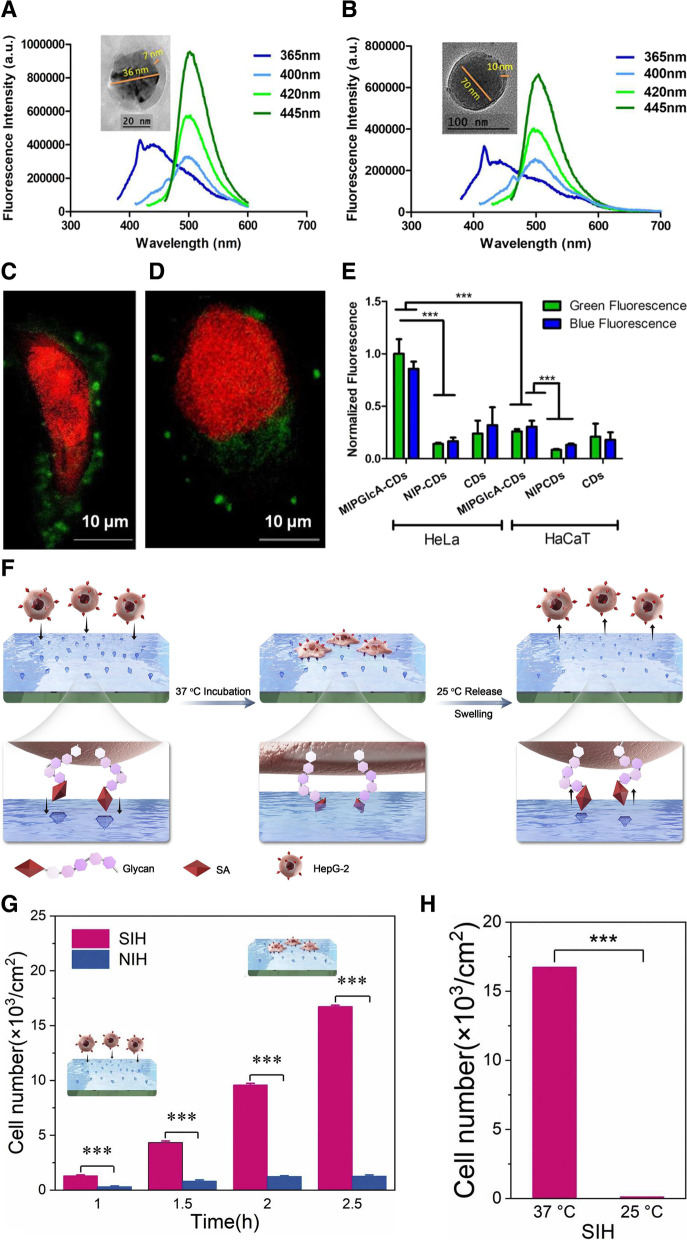
**A** CD-MIPGlcA and (**B**) CD-NIP with different excitation wavelengths in the range of 365–445 nm, slit 2.5 nm. The small peak around 420 nm at excitation 365 nm is due to the water Raman peak. Insets are corresponding transmission electron microscopy (TEM) images. Confocal micrographs showing labeling of GlcA on a single (**C**) HeLa and (**D**) HaCaT cell by CD-MIPGlcA (green) and nuclear staining with propidium iodide (red). **E** Analysis of labeled cells with CD-MIPGlcA, CD-NIP, and CD as obtained from ImageJ by measuring the normalized fluorescence of each single cell area from five different images. **F** Thermo-responsive SA-imprinted hydrogel layer enables selective capture and release of cancer cells. **G** The numbers of captured HepG-2 at different time intervals. **H** Comparison of capture and release efficiency of cancer cells. **A**, **B**, **C**, **D**, **E** Reproduced with permission from [114], published by The American Chemical Society 2018. **F**, **G**, **H** Reproduced with permission from [58], published by Elsevier 2021

The detection of cancer cells is one of the biggest challenges in modern science. Only a limited number of surface proteins can be used as targets, and more often, it is about the ratiometric expression of those targets rather than their presence or absence that differentiates a healthy cell from a cancer cell. Demir et al. devised a simple fluorescent probe for the targeting and imaging of cancer cells (Fig. 8A-E) [114]. The hydrothermal synthesis of N-doped carbon nanodots (CDs) from starch and L-tryptophan had a 25.1 ± 2% yield with an average particle size of 3.2 nm. The QDs were quoted with a mixture of functional monomers, AB and methacrylamide as well as a cross-linker, ethylene glycol dimethacrylate (EGDMA) to molecularly imprint a shell of glucuronic acid cavities. As the QDs emitted fluorescence at 450 nm, internal light was used to polymerize the MIP. A comparison between human cervical cancer cell staining and reference human keratinocytes demonstrated the functionality of the probe.

Sialic acid (SA) is a nine-carbon atom sugar that is highly present on the surface of cancer cells at the glycan terminal of membrane proteins and lipids. Ma et al. devised a thermo-responsive imprinted hydrogel layer for the detection of SA (Fig. 8F-H) [58]. To overcome the challenges presented by the commonly used lectin-based detection technique that can be expensive and easily denatured, the imprinting process was performed at 37 °C using a thermo-responsive functional monomer. Interestingly, this feature allows for the capture and release of cancer cells in both culture media and real blood samples. Beyer et al. also focused on the detection of SA [115]. The team developed an SA-imprinted fluorescent core–shell sensing probe. In brief, a mixture of methacrylamide, 2-{3-(4-nitrobenzo[c] [1,2,5] oxadiazol-7-yl)ureido} ethyl methacrylate, vinylbenzene boronic acid, ethylene glycol dimethacrylate, and N,N-dimethylformamide served as the pre-polymerization solution for monosaccharide imprinting. Silica-coated polystyrene particles, SA, and an initiator, 2,2′-azobis(2,4-dimethylvaleronitril), were added to the solution to complete the reaction. Interestingly, after removal of the template, the probe was tested on multiple cancer cell lines with pentavalent SA conjugates, and the results demonstrated different binding patterns according to the level of α2,3- and α2,6-SA expression. Targeting, isolation, and imaging are great applications of MIPs. However, despite these promising results in cancer cell detection, we are still far from the efficient sensing of circulating cancer cells. Further development of molecular imprinting strategies will shed new light on this issue (Table 2).

**Table 2:** Molecularly imprinted polymer (MIP)-based cancer biomarker detection

Biomarker	MIP^a^ components	Template	Mechanism	Detection range	LOD	Reference
Nucleic acid	Monomer: Methacrylic acid (MAA) Additive: Nafion	Rhodamine B	Electrochemical sensing	10 fM—100 nM	2.53 fM	[103]
	Monomer: (3-aminopropyl)triethoxysilane (APTES) Crosslinker: Tetraethyl orthosilicate (TEOS)	Malachite green (MG)	Phosphorescence and fluorescence sensing	0.089 -1.79 μg/mL	19.48 ng/mL	[102]
	Monomer: Dopamine	MicroRNA 21	RNA separation	-	-	[101]
	Monomers: acrylamide (AA) and N,N′-methylenebisacrylamide (MBAm) Initiator: ammonium persulfate (APS) Catalyst: N,N,N′,N′-tetramethylethylenediamine (TEMED)	HeLa cell-derived ribosomes	Ribosome and associated RNA separation	-	-	[116]
Protein	Monomer: 3, 4-ethylenedioxythiophene	Human epidermal growth factor receptor 2 (HER2)	Electrochemical sensing	1—200 ng/mL	0.43 ng/mL	[104]
	Monomer: APTES, 3-ureidopropyl-triethoxysilane, isobutyltriethoxysilane, and tetraethyl orthosilicate (TEOS)	Carcinomaembryonic antigen (CEA)	Surface enhanced Raman spectroscopy	-	5.6 × 10^−14^ M	[106]
	Monomer: Dopamine	Carcinomaembryonic antigen (CEA)	Electrochemical sensing	0.001—1000 ng/mL	0.2589 pg/mL	[97]
			Surface enhanced Raman spectroscopy	0.1 pg/mL- 10 μg/mL	0.064 pg/mL	[105]
		Prostate-specific antigen (PSA)	Electrochemiluminescence	5 pg/mL—50 ng/mL	3.0 pg/mL	[107]
	Monomer: Acrylamide Crosslinker: N,N′-methylenebisacrylamide	Prostate-specific antigen (PSA)	Electrochemical impedance spectroscopy	0.01—100 ng/mL	5.4 pg/mL	[108]
		myoglobin (Myo)		1—20,000 ng/mL	0.83 ng/mL	
Exosome	Monomer: 2-methacryloyloxyethyl phosphorylcholine	Exosomes	Fluorescence sensing	-	6 pg/mL	[112]
		Silica nanoparticles		-	1.2 × 10^–17^ M	[113]
	Monomer: MAA and 4-vinylphenylboronic (VPBA)	Silica nanoparticles wrapped by horseradish peroxidase with diameters in 50, 100, and 150 nm	Electrochemical sensing	-	1.44 × 10^3^, 5.68 × 10^2^ and 7.70 × 10^2^ particles/mL	[110]
	Monomer: 2-(diethylamino)ethyl acrylate and acrylamide Cross-linker: N,N′-methylene diacrylamide	Silica nanoparticles	Exosome capture/proteome analysis	-	-	[111]
	Monomer: Acrylamide (AAm), N, N-methylene bisacrylamide (MBA), N-isopropylacrylamide (NIPAAm), and MAA Initiator: N,N,N′,N′-tetramethylethylenediamine (TEMED) and ammonium persulfate (APS)	Exosomes	“Turn-on” fluorescence sensing	-	1.3 × 10^6^ particles/mL	[94]
			Fluorescence resonance energy transfer		2.43 × 10^6^ particles/mL	[95]
Cell	Monomer: 4-acrylamidophenyl)(amino)methaniminium acetate (AB) and methacrylamide Initiator: Coumarin 6 and triethylamine Cross-linker: Ethylene glycol dimethacrylate (EGDMA)	Glucuronic acid	Fluorescence imaging	-	-	[114]
	Monomer: Acrylamide (AAm), N-(3-aminopropyl)methacrylamide hydrochloride (NAPMAAm), N-isopropylacrylamide (NIPAAm), and 3-methacrylamidophenylboronic acid Initiator: N,N,N′,N′-tetramethylethylenediamine (TEMED) and ammonium persulfate (APS) Cross-linker: N,N′-methylenebisacrylamide (MBAAm)	Sialic acid (SA)	Cancer cell capture	-	-	[58]
	Monomer: methacrylamide (MAAm), 2-{3-(4-nitrobenzo[c][1,2,5]oxadiazol-7-yl)ureido} ethyl methacrylate, vinylbenzene boronic acid (VBBA) and EGDMA Initiator: 2,2′-azobis(2,4-dimethylvaleronitril) (ABDV)		Fluorescence imaging	-	-	[115]

^a^Molecularly imprinted polymer

## MIP-based cancer therapy

Cancer is the second leading cause of death and a major public health issue worldwide [117]. Millions of people are diagnosed with cancer annually. There are many things that should be considered in cancer treatment, including multidrug resistance issues, intrinsic complexity and heterogeneity of tumors, and the selective ability of anticancer therapeutics to only target cancer lesions [118–120]. Despite these factors playing an important role in cancer therapy, the most important thing to consider is that the drug is only effective in certain places. Conventional therapeutic agents have several limitations that affect their efficacy and safety profile, resulting in permanent genetic alterations and adverse effects [93, 121] owing to their non-specific targeting ability. These drugs can damage unrelated healthy tissue and cannot deliver sufficient amounts to target sites. MIPs solve these problems by targeting certain proteins such as saccharides and glycans, which are well-known biomarkers over-expressed and secreted by cancer cells [12]. This property of MIPs can be utilized in drug delivery, PTT and PDT, as well as a therapeutic in its own right.

### Drug delivery

Drug delivery systems based on NPs can be engineered to load anticancer drugs in a variety of configurations and selectively reach cancer sites while avoiding healthy tissues [122]. In cancer treatment, chemotherapy remains the prevailing course for many types of cancers because of its high efficiency compared to other methods [123, 124]. However, chemotherapeutics, when used alone, lack specificity and selectivity, resulting in damage to healthy tissues, rapid clearance by the gastrointestinal tract, thus leading to severely diminished therapeutic effectiveness. Among the advanced nanocarriers for targeted delivery, MIPs have shown a breakthrough in overcoming the aforementioned challenges with high affinity and selectivity, easy fabrication process, low cost, and excellent stability. Recently, their potential has led to a rapid expansion from the traditional separation and purification fields to the expanding field of tumor-targeted drug delivery [125]. Canfarotta et al. produced novel double-imprinted nanoMIPs based on a solid-phase method [126]. MIPs were loaded with doxorubicin (DOX) and targeted toward the linear epitope of epidermal growth factor receptors (EGFRs). EGFRs are overexpressed in several malignancies and are one of the critical regulators of cell proliferation and cancer invasiveness. EGFR-nanoMIPs measuring 150–200 nm exhibited the highest binding affinity toward breast cancer MDA-MB-468 cells overexpressing EGFR but did not show any appreciable binding affinity toward SKBR3 normal cells. Upon interaction with EGFR-nanoMIPs, EGFR undergoes endocytosis and subsequent internalization, resulting in its accumulation in the cytoplasm. These results demonstrate that DOX-loaded EGFR-nanoMIPs lead to the preferential killing of cells that overexpress EGFR. Apart from chemotherapy, MIPs have shown potential for the delivery of various new anticancer therapeutic agents, including antisense oligonucleotides, small interfering RNA, mRNA, and DNA inhibitor oligonucleotides [127].

**Fig. 9:**
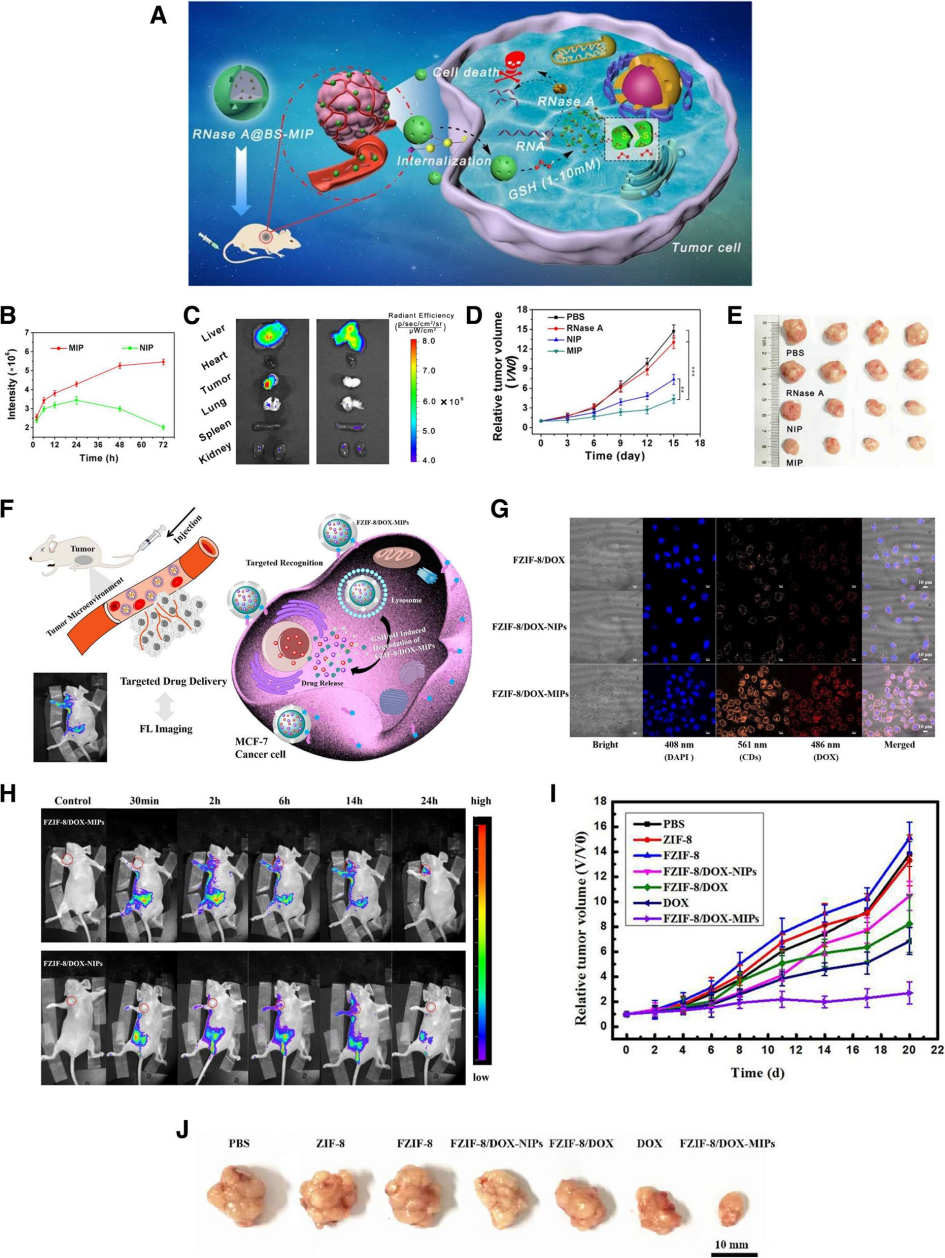
**A** Schematic illustration of drug delivery route of RNase A@biodegradable silica nanoparticles (BS-NPs) through redox-triggered biodegradation. **B** Time-dependent variation of fluorescence intensity at tumor sites of HepG2 tumor-bearing mice. **C** Ex vivo fluorescence imaging of tumor and other organs collected from mice in the sialic acid (SA)-imprinted molecularly imprinted polymer (MIP) and nonimprinted (NIP) group after 72 h. **D** Relative tumor volume growth curves of the mice during the 15 d of treatment. **E** Photograph of the dissected tumors in each treated group. **F** Schematic representation of targeted fluorescent imaging and glutathione (GSH)/pH-responsive drug delivery route of FZIF-8/DOX-MIPs. **G** Confocal microscopy images of MCF-7 cancer cells after coculture with FZIF-8/DOX-MIPs for 1 h. **H** Fluorescence images of MCF-7 tumor-bearing mice with at different times after intraperitoneal injection of MIPs and NIPs. **I** Relative tumor volume and (**J**) representative photographs of excised tumors in different groups after 20 d of treatment. **A**, **B**, **C**, **D**, **E** Reproduced with permission from [93], published by American Chemical Society 2021. **F**, **G**, **H**, **I**, **J** Reproduced with permission from [131], published by American Chemical Society 2020

Recently, stimulus-responsive MIPs have been developed via the introduction of certain functional monomers into their structures to enhance the efficacy of drug delivery. These smart nanoMIPs can release drugs on demand, allowing a more controlled drug delivery process [62, 125]. In response to an intrinsic stimulus, such as pH levels, enzymes, and reducing agents, or extrinsic stimuli, such as heat and electromagnetic fields [128], MIPs can achieve good control of release kinetics. As an indicator for some cancer cells, SA has been attracting intense interest for compounds with the cis-diol structure to covalently combine or dissociate with phenyl-boronic acid groups [129, 130]. Lu et al. reported SA-imprinted biodegradable silica NPs (BS-NPs) for therapeutic protein delivery enabling targeted cancer therapy [93]. BS-NPs could specifically bind SA, which is overexpressed at the outermost position of glycoproteins on the cancer cell membrane, and be subsequently absorbed into the cell through endocytosis and degraded in the intracellular acidic microenvironment, resulting in a progressive cargo accumulation profile and enhanced cell cytotoxicity. In comparison to NIPs, BS-NPs showed superior targeting ability and enhanced cytotoxicity against SA-overexpressing human liver carcinoma HepG2 cells (Fig. 9A-E). In vitro, under a reductive tumor microenvironment (pH 4.5–5.5), the BS-NPs disintegrated into small fragments within 48 h. These results show the promising potential of the BS-NP-based nanoplatform for controlled drug delivery.

Intrinsic stimuli are relatively complex, uncontrollable, and present variations between preclinical and clinical models [128, 132]. Extrinsic stimuli can be controlled more accurately, leading to the development of numerous smart nanomaterials for drug delivery systems. Typical extrinsic stimuli that are manipulated from outside the body are light, temperature, ultrasound, electric pulses, and magnetic field. Liu et al. fabricated an NIR-light-responsive surface MIP (NSMIP) using upconversion NPs as the core, a green-light-responsive azobenzene derivative as the functional monomer, and paracetamol as the template [133]. The NSMIP showed a controlled release of the drug in aqueous solution and through porcine tissue under laser irradiation (980 nm, 5 W/cm^2^). Unlike MIPs that display single-response behavior, those with dual or multiple responses exhibit multifunctionality and a heightened degree of responsiveness [134]. Kubo et al. reported a new thermal-responsive drug delivery system using Fe_3_O_4_ NPs coated with MIPs [135]. The Fe_3_O_4_ was used as a magnetic-field stimuli-sensitive seed (MTS) that generates heat under an alternating current (AC) magnetic field, and MAA was used as a thermal-responsive MIP layer for effective drug release at 60 ℃, which was sufficient to cleave most of the interactions with the target molecule, methotrexate (MTX), which was used as an anticancer drug. MIP-MTS showed a higher amount of MTX adsorption than NIP-MTS, and MTX was completely released within 10 min at 60 ℃, but not at 25 ℃. These MIPs have achieved controlled drug release by applying an AC magnetic field using the heat-generating property of MTS. Another study, Li et al. also, prepared temperature and magnetism bi-responsive molecularly imprinted polymers (TM-MIPs) based on Fe_3_O_4_ encapsulating carbon nanospheres [136]. TM-MIPs were prepared by grafting N-isopropylacrylamide (NIPAM) as the thermosensitive monomer on a surface of Fe3O4 modified with MPS, followed adding methylene bisacrylamide (MBA) as a crosslinker to form an imprinting layer via free radical polymerization. 5-Fluorouracil (5-FU), which widely used in the clinical treatment as an anticancer drug, was used as a target template. TM-MIPs showed the potential for drug delivery systems by verifying temperature-controlled absorption and release behavior. Future research aimed at achieving more effective and controlled drug delivery can be facilitated through the development of MIPs capable of targeting multiple targets and exhibiting responses to multiple stimuli.

To monitor the treatment response, it is very important to directly visualize the release and accumulation of the drug to specific sites during the delivery process. The integration of treatment and diagnosis, defined as theragnostics, is being extensively utilized as imaging-guided drug delivery systems for cancer treatment [137]. Among the various theragnostic nanocarriers, MIPs, in particular, have a great advantage in that there is no need to modify the surface with ligands and they already contain specific binding sites. In addition, because therapeutics can be encapsulated directly into the MIP network during its synthesis, they can be protected until the cleavage of the carrier [12]. Qin et al. synthesized a fluorescent zeolitic imidazolate framework-8 loaded with doxorubicin (FZIF-8/DOX) as a core and prepared MIP with the epitope of CD59 cell membrane glycoprotein as a template, termed FZIF-8/DOX-MIPs [131]. These FZIF-8/DOX-MIPs not only targeted tumors that overexpressed CD59 glycoproteins, but also allowed for both the in vitro and in vivo CD-based fluorescence imaging of cancer cells (Fig. 9F-J). Consequently, MIP-coated FZIF-8/DOX can be degraded in the tumor microenvironment, and FZIF-8/DOX is further degraded in response to the weakly acidic conditions, resulting in controllable release of DOX for targeted treatment. A wide range of sources, such as QDs, CDs, and various dyes, have been extensively applied in the biological field for imaging [27]. In recent years, compared to fluorescent dyes, silicon NPs (SiNPs) have been increasingly used in image-guided drug delivery owing to their excellent optical properties, low biotoxicity, and biocompatibility [138].

### Photothermal therapy

PTT uses photothermal agents that generate heat upon irradiation with a specific wavelength of light, thus increasing the temperature of cancerous tissues and inducing cell death. Over the past few years, PTT has emerged as an attractive therapeutic in oncology because it offers several advantages over traditional treatments, including spatially controlled action, noninvasiveness, and low toxicity [139, 140]. In recent years, near-infrared (NIR) light-mediated PTT has been widely used in cancer therapy, which can cause apoptosis or necrosis of cancer cells by triggering a localized hyperthermia effect [141–143]. The photothermal agents generally used in PTT can be classified into four main subgroups: plasmon resonance-generating metallic nanostructures, carbon-based light-absorbing materials, organic materials, and polymeric materials [144]. However, the lack of specificity of nanomaterials can result in damage to the surrounding healthy cells. To solve this problem, targeting biomolecules such as antibodies, aptamers, peptides, and receptors are commonly conjugated to nanomaterials. However, these biomaterials sometimes suffer from poor specificity, stability, and reproducibility. MIPs, which are robust biomimetic recognition molecules, exhibit many advantages, such as simple preparation, chemical stability, and high specificity toward template molecules. Clearly, combining these elements is highly favorable for more efficient cancer-targeting PTT.

Wang et al. reported sialic acid-imprinted upconversion NPs (UCPs@MIPs) as artificial antibodies for active tumor targeting and microinvasive PTT [145]. UCPs@MIPs were prepared using boronic acid-functionalized lanthanide-doped UCPs as a substrate, followed by the formation of an imprinting layer via the in-water copolymerization of dopamine and m-aminophenylboronic acid synchronously acting as a photothermal couplant and tumor-targeting element. In vitro, confocal microscopy demonstrated that UCPs@MIPs enabled not only the distinction between SA-overexpressing cancerous cell lines (HePG2 and MCF-7) and normal cell lines (L02 and MCF-10A), but also complex tissue levels. In addition, tumor cells underwent dramatic cell death as a consequence of the heat generated by 980 nm laser radiation, while normal cells maintained a remarkably high viability (> 85%). In vivo, UCPs@MIPs inhibited the proliferation of HepG2 tumors in mice within 20 d based on the fast PTT mode with a single treatment time at the second level. As designed, UCPs@MIPs may be used to achieve tumor-targeted microinvasive PTT by semi-accurate hyperthermia tumor ablation while protecting normal tissue from overheating.

**Fig. 10:**
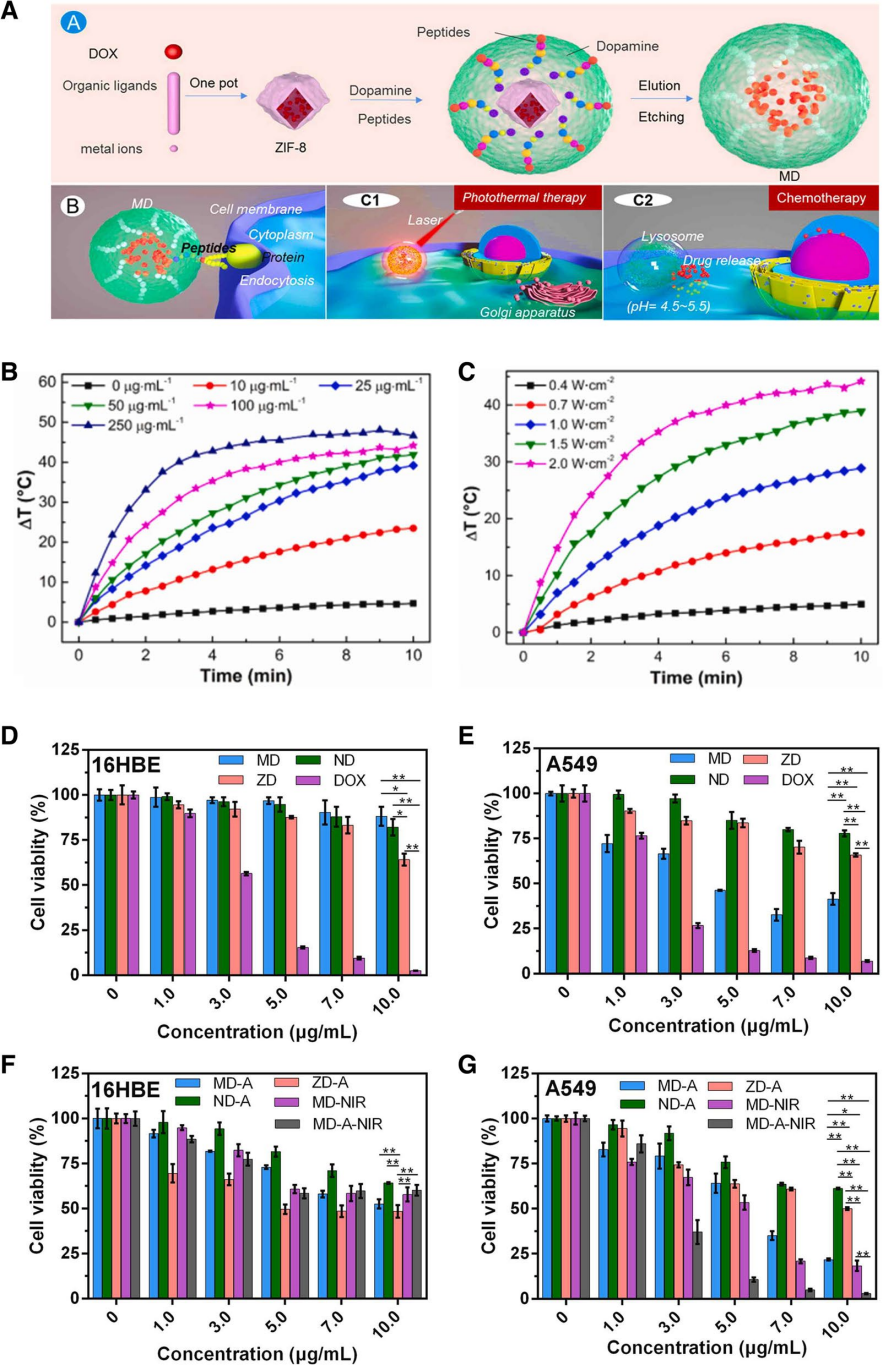
**A** Synthesis process of capsule-like molecularly imprinted polymers (MIPs) nanoparticles (NPs) as drug carriers with chemo-photothermal therapeutic effects. Photothermal heating curves of the MIP water solution (**B**) with various concentrations and (**C**) various power density under 808 nm laser irradiation. **D** Cell viabilities of 16HBE cells incubated with doxorubicin (DOX)@MIP (MD), DOX@NIP (ND), zeoliticimidazolate framework-8 (ZIF-8)@DOX (ZD), and DOX at pH = 7.4 (MD, ND, ZD, DOX), **E** pH = 5.0 (MD-A, ND-A, ZD-A), and with near-infrared (NIR) laser irradiation (MD-NIR, MD-A-NIR) (808 nm, 1.5 W/cm^2^, 5 min). **F** Cell viabilities of A549 cells incubated with MD, ND, ZD, DOX at pH = 7.4 (MD, ND, ZD, DOX), **G** pH = 5.0 (MD-A, ND-A, ZD-A), and with NIR laser irradiation (MD-NIR, MD-A-NIR) (808 nm, 1.5 W/cm.^2^, 5 min). The cells were pretreated with acidic PBS (pH = 5.0, denoted as X-A). X represents MD, ND or ZD. **A**, **B**, **C**, **D**, **E**, **F**, **G** Reproduced with permission from [153], published by Elsevier 2021

Compared to a single treatment, integrating PTT and chemotherapy reduces the regrowth of residual tumors by enhancing the therapeutic coverage and improving the therapeutic index [146, 147]. The heat generated by PTT increases cell membrane penetrability, resulting in enhanced drug uptake by cancer cells or the increased cytotoxicity of certain heat-sensitive anticancer drugs [148, 149]. Xu et al. presented a novel controllable drug delivery system based on this photothermal effect [150]. Antibacterial polymerizable imidazolium-based ionic liquids were used as surfactants in mini emulsion polymerization. During polymerization, graphene quantum dots were encapsulated with DOX in MIPs. After irradiating with an 808 nm laser for 40 min, the MIP was increased to 43 ℃; because of this, the interactions with the CQDs, imprinting layer, and DOX were weakened, and the drug was gradually released for 3 h, showing that the cumulative release was slowly increased to 36.54%. Drug-loaded MIPs combined with inductive NIR heating could be applied in synergy with chemotherapy and thermotherapy for cancer therapy. Polymerizable DA has frequently been used as a functional monomer and crosslinker of MIPs owing to its excellent biocompatibility, biodegradability, and photothermal conversion ability [151, 152]. Considering this, Liu et al. designed a simple capsule-like MIP for targeted chemo-photothermal synergistic cancer therapy [153]. Using zeolitic imidazolate framework-8 (ZIF-8) carrying DOX as the core, polymerizable DA as the functional monomer to form the MIP layer as well as a photothermal agent, and an epitope of EGFR as a template molecule, DOX@MIP (MD) was constructed to specifically bind EFGR-overexpressing cancer cells. After irradiating the MDs with an NIR laser (808 nm) for 10 min, the temperature rapidly increased to 44.2 ℃, with a photothermal conversion efficiency of 36.3%. These MDs specifically targeted EGFR-overexpressing cancer cells (A549 and MDA-MB-468) while avoiding normal cells (16HBE). The MIP layer maintained a rigid shape under physiological conditions but was degraded by the acidic tumor environment, releasing DOX. Taken together, MD NPs may be used as chemo-photothermal therapy tools with minimal toxicity to normal cells (Fig. 10A-G).

Additionally, PTT can also be combined with immunotherapy for better cancer treatment. PTT, when combined with immunotherapeutic components, can activate immune stimulation by generating heat within the tumor microenvironment leading to tumor regression [154–156]. Aiming for the destruction of tumors through the synergic effect of PPT and immunotherapy, Ma et al. fabricated human serum albumin (HSA)-imprinted polymer-coated Fe_3_O_4_ NPs (Fe_3_O_4_@MIPs) through the oxidative polymerization of polydopamine in the presence of HSA [157]. The MIP exhibited rapid and specific HSA reabsorption with an improved photothermal effect of the Fe_3_O_4_ NPs. Albumin binds to the secreted protein acidic and rich in cysteine (SPARC), which is overexpressed in most tumor tissues. Compared to the non-albumin-imprinted particles (Fe_3_O_4_@NIPs) in vitro, the HAS-imprinted Fe_3_O_4_@MIPs could specifically bind to 4T1 cells (SPARC^+^ cells) and resulted in less elimination from RAW 264.7 cells, which indicates the involvement of the reticuloendothelial system. In vivo, albumin camouflage in Fe_3_O_4_@MIPs led to a 2.6-fold improvement in tumor accumulation in comparison to Fe_3_O_4_@NIPs, and more heat was produced upon 808 nm laser irradiation, which further triggered efficient immunogenic cell death. In addition, combining the programmed death-ligand 1 (PD-L1) antibody with Fe_3_O_4_@MIPs PTT could effectively inhibit not only the growth of primary tumors, but also tumor metastasis by eliciting immunological effects.

### Photodynamic therapy

In contrast to PTT, which requires photothermal conversion agents to generate heat for the thermal ablation of cancer cells, PDT is another powerful phototherapeutic strategy to treat cancer. PDT uses three major elements: light, a photosensitizer (PS), and oxygen. Under certain light irradiation conditions, the PS becomes activated from a ground singlet state to an excited triplet state and then undergoes two different photochemical reaction mechanisms to produce highly toxic ROS [158, 159]. The type I reaction mechanism involves electron/hydrogen transfer directly from the PS to the biomolecules, yielding free radicals or radical ions, which react with molecular oxygen to produce ROS such as hydrogen peroxide, superoxide anions, and hydroxyl radicals. In contrast, the type II reaction involves direct energy transfer from the triplet state PS to molecular oxygen, forming another ROS, an extremely electrophilic singlet oxygen [160]. PDT has received much attention over the years as an emerging anticancer therapeutic modality owing to its numerous merits, including noninvasiveness, selective localized irradiation, and minor side effects [161].

**Fig. 11:**
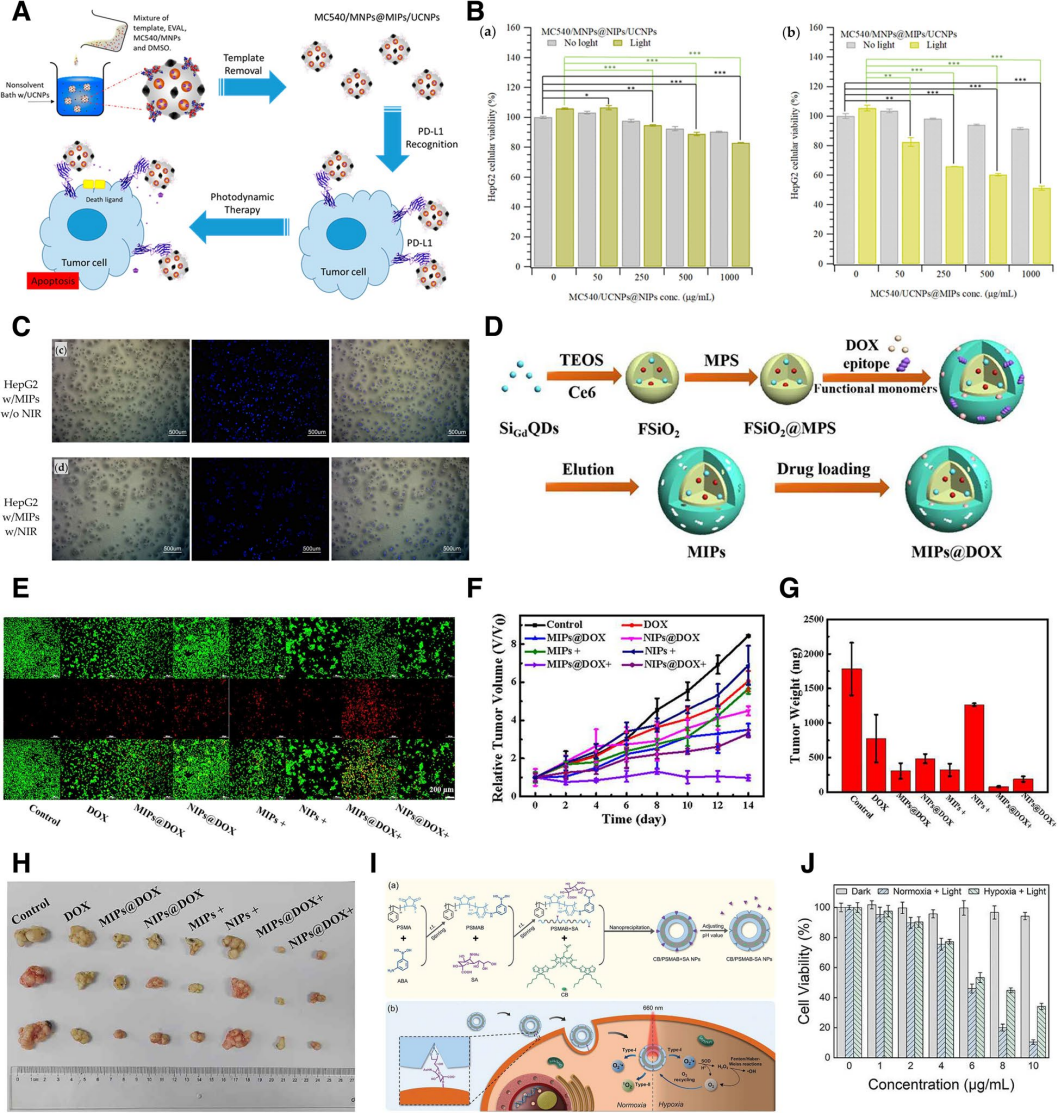
**A** Preparation procedure and therapeutic mechanism of programmed death-ligand 1 (PD-L1) peptide-imprinted composite nanoparticles (NPs). **B** Cellular viabilities of HePG2 cells incubated with various concentrations of non-imprinted polymers (NIPs) and molecularly imprinted polymers (MIPs). **C** Optical, 4′,6-diamidino-2-phenylindole (DAPI)-stained, and merged images of HepG2 cells treated with MIPs without and with NIR irradiation. **D** Schematic illustration for the preparation of MIPs@doxorubicin (DOX). **E** Live/dead cell staining assays under various treatments. **F** Relative tumor volume in 14 d with various treatments. The average tumor weight (**G**) and corresponding tumor tissues (**H**) after treatments for 14 d. **I** Synthetic procedure and photodynamic killing mechanism of two cyclopenta-dithiophene units and one boron dipyrromethene core as a photosensitizer, and poly(styrene-co-maleic anhydride) (PSMA) modified with phenylboronic acid as a polymer matrix (CB/PSMAB-SA NPs) toward sialic acid (SA) over-expressed cancer cells. **J** Cellular cytotoxicity of DU 145 cells after treatment with MIPs under normoxia or hypoxia along with light irradiation (660 nm, 0.1 W/cm.^2^). **A**, **B**, **C** Reproduced with permission from [163], published by MDPI 2021. **D**, **E**, **F**, **G**, **H** Reproduced with permission from [162], published by American Chemical Society 2020. **I**, **J** Reproduced with permission from [164], published by Wiley 2022

There are still several limitations to overcome for effective PDT. Owing to their hydrophobic nature, easy aggregation, and low payloads, PSs possess poor accumulation and tumor-targeting capabilities, leading to an unsatisfactory therapeutic effect [162]. Among the different nanomaterial-based PS-loading carriers, MIPs have recently received attention owing to their high target specificity and ease of modification. PSs can be physically loaded or chemically conjugated to nanocarriers, leading to sufficient accumulation at the tumor site. Thus, this process achieves efficient PDT without affecting healthy tissue. Lin et al. incorporated merocyanine 540 (MC540)-grafted magnetic NPs into MIPs, where the imprinting layer was formed by poly(ethylene-co-vinyl alcohol) (EVAL) via a precipitation method, and entrapped UCNPs in the imprinted particles for exciting the MC540, resulting in catalyzing the generation of ROS [163]. The PD-L1 peptide sequence (EDLKVQHSSYRQRA) was used as a template to enhance the targeting ability of MIPs against HepG2 human liver cancer cells. In vitro studies showed that upon irradiation with 980 nm NIR for 5 min, MIPs at a concentration of 1.0 mg/mL induced apoptosis in about half of the cells; however, the viability of HepG2 cells was maintained at about 85%. In addition, imaging of HepG2 cells confirmed that laser irradiation of MIPs induced a decrease in cell survival by about 1.7 times compared to that of non-irradiated MIPs (Fig. 11A-C).

Evidence from recent literature suggests that when administered as a monotherapy in vivo, PDT may lead to cancer cell resistance by inducing hypoxia, thus seriously hindering the therapeutic effect [165, 166]. However, when used in combination with other therapeutic methods PDT can significantly inhibit tumors and prevent resistance [167]. Thus, developing a multifunctional nano-system is an efficient method for cancer treatment. Peng et al. prepared dual-template imprinted polymers with a core–shell structure for synergistic chemo-/photodynamic cancer therapy [162]. Fluorescent silica NPs (FSiO_2_) were used as a core in which gadolinium-doped silicon QDs for target fluorescent/magnetic resonance dual imaging, combined with chlorin e6 (Ce6) as a PS, were encapsulated. The imprinted layer was constructed on the surface of FSiO_2_ via free-radical polymerization using the epitope of CD59 (YNCPNPTADCK) and DOX as a dual template (Fig. 11D-H). The resulting DOX-loaded MIPs (MIPs@DOX) exhibited a specific targeting ability for CD59-overexpressing MCF7 cancer cells with FI-guided target imaging. After binding with cancer cells, DOX was effectively released from MIPs@DOX in the acidic microenvironment, integrating with a high abundance of cytotoxic ^1^O_2_ generated from 655 nm light irradiation by Ce6. Cell imaging and synergistic targeted therapy were successfully achieved both in vitro and in vivo with negligible toxicity toward healthy tissues and organs.

Another option to overcome hypoxia and increase the efficiency of PDT involves changing the PS agent. Most traditional PSs function through the type II mechanism, which requires an adequate level of O_2_. However, the proliferation of aberrant neoplastic cells and the distortion of tumor vasculatures result in unexpected local hypoxia, which is worsened by the consumption of oxygen during PDT. To address this problem, Peng et al. developed conjugated oligomer-based hollow NPs imprinted with SA for targeted photodynamic therapy under hypoxia [164]. The MIPs were fabricated using a conjugated oligomer, which consisted of two cyclopenta-dithiophene units and one boron dipyrromethene core as a photosensitizer, and poly(styrene-co-maleic anhydride) (PSMA) modified with phenylboronic acid as a polymer matrix, termed CB/PSMAB-SA NPs. Owing to sialic imprinting, it was confirmed that CB/PSMAB-SA NPs could selectively bind toward SA over-expressed human prostate carcinoma cells (DU 145) and efficiently accumulate in the cells compared to human cervical cancer cells (HeLa) with low expression levels of SA. In vitro experiments demonstrated that CB/PSMAB-SA NPs had an efficient photodynamic performance by generating prominent ^1^O_2_ via the type II mechanism under normoxia (20% O_2_) and O_2_^ㅡ^• via the type I mechanism even under a severely hypoxic environment (1% O_2_) triggered by 660 nm laser irradiation (Fig. 11I, J).

### Biological activity regulation

**Fig. 12:**
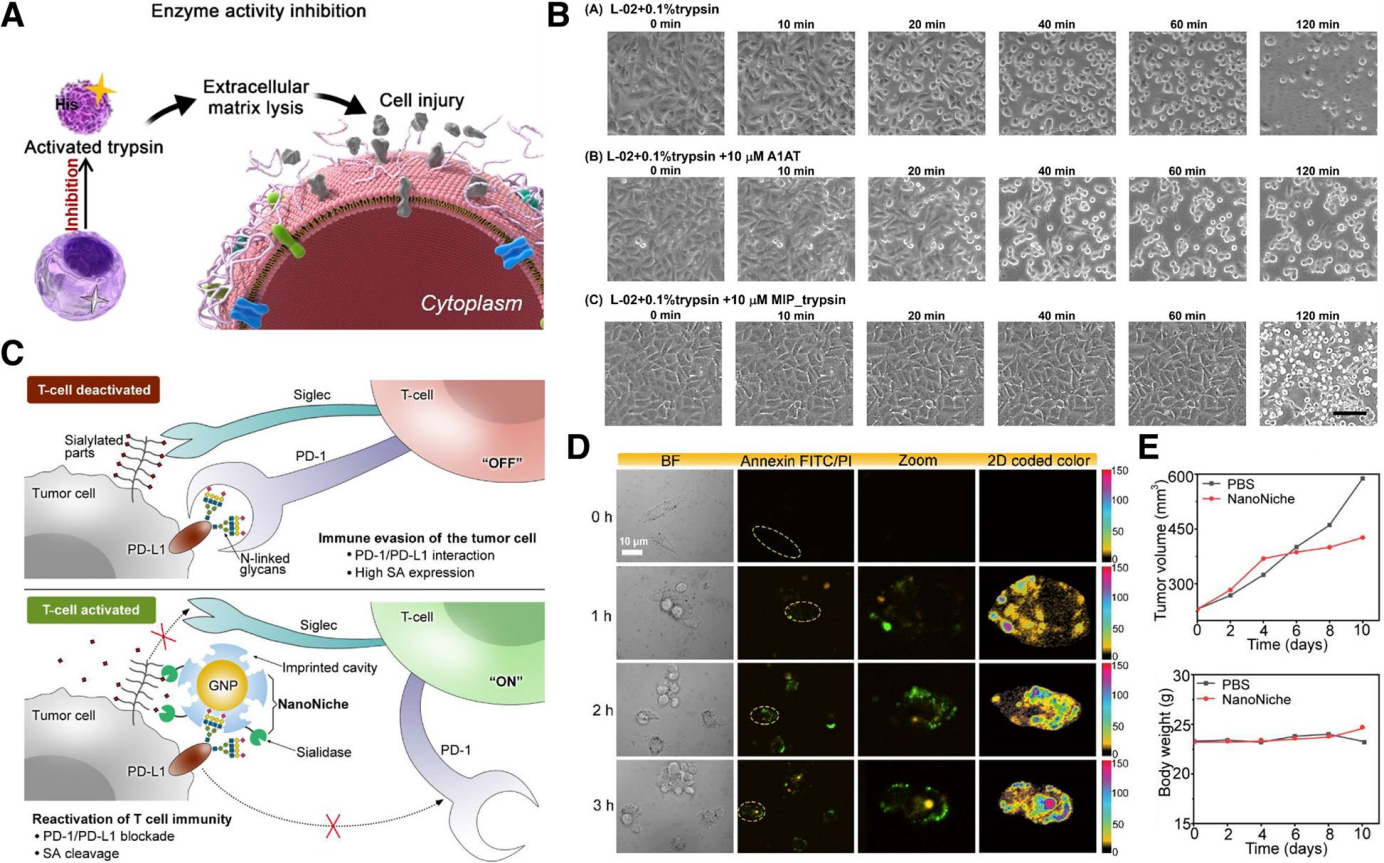
**A** Schematic illustration of molecularly imprinted polymers (MIP)_trypsin used as an enzyme inhibitor to prevent trypsin-caused cell damage. **B** Microscopy imaging of L-02 cells incubated with trypsin or trypsin/A1AT or trypsin/MIP_trypsin. **C** Graphical illustration of the principle of tumor cell immune evasion from T-cells and the reactivation of T-cell immunity by blocking programmed death-ligand 1 (PD-L1) via N-glycan-imprinted NanoNiche with a desialylation function. **D** Microscopy images of the T-cell-mediated apoptotic effect in MDA-MB-231 cells incubated with NanoNiche (220 μL) after treatment for 0, 1, 2, and 3 h. **E** Average tumor volumes and body weights of tumor-bearing mice under different treatments at different time intervals. **A**, **B** Reproduced with permission from [173], published by Wiley 2021. **C**, **D**, **E** Reproduced with permission from [176], published by American Chemical Society 2021

MIPs can be used as drugs by regulating proper biological activity. The design and synthesis of potent enzyme inhibitors have recently received considerable attention owing to their great potential in regulating biological processes through enzyme inhibition or activation [168, 169]. Small organic molecules, with easy large-scale production through chemical synthesis, are commonly used as inhibitors. However, they exhibit side effects owing to their lack of specificity. Antibodies have been proposed as an alternative; however, they are expensive and unstable in physiological environments. To address the above issues, the molecular imprinting strategy has gained considerable attention as a viable option because it has proven to be a highly tailored artificial inhibitor with high affinity and selectivity toward various target enzymes [170, 171]. Among the various enzymes, trypsin, one of the best-characterized matrix serine proteases, plays essential roles in various pathological processes, including tumor invasion and metastasis [172]. The inhibition of trypsin activity offers an appropriate treatment for active trypsin-dependent cell injury. Xu et al. developed molecular imprinting enzyme inhibitors with high specificity and potent inhibitory effects toward trypsin [173]. The trypsin-imprinted NPs (MIP_trypsin) were obtained by immobilizing the enzyme on glass beads, which were functionalized with IDA-Cu^2+^, followed by the formation of an imprinting cavity employing solid-phase imprinting. With this method, the fabricated MIP_trypsin could obtain an exposed immobilized active site of the enzyme, which made it possible to obtain oriented binding sites at the surface. MIP_trypsin showed high selectivity toward trypsin through a quartz crystal microbalance sensor immobilized with proteins that have similar properties as trypsin. In addition, an inhibitory effect toward trypsin was observed, with an inhibition constant of 3.4 nM, whereas no inhibitory effect was observed for kallikrein, which has a 70.3% sequence similarity with trypsin. In vitro assays revealed that MIP-trypsin could protect human normal liver cells (L-02) from active trypsin-caused cell damage by preventing tryptic digestion-induced extracellular matrix lysis (Fig. 12A, B). Dihydrofolate reductase (DHFR) has been studied for the treatment of a wide variety of human diseases owing to its essential role in DNA synthesis [174]. Blocking the enzymatic activity of DHFR in tumor cells can inhibit DNA synthesis and ultimately lead to cell death. Qin et al. proposed a new anti-metabolic therapy using molecularly imprinted NPs with an inhibitory effect on the enzymatic activity of DHFR to inhibit tumor growth [175]. MIP-(3-propanecarboxyl) triphenyl phosphonium bromide (CTPB) was prepared by imprinting the active center peptide of DHFR related to DNA metabolism, followed by modifying the mitochondrial targeting moiety (CTPB) on the surface of MIP for effective targeting of the mitochondria. MIP-CTPB exhibited excellent binding specificity with DHFR and blocked its catalytic activity in a dose-dependent manner. MIP-CPPB showed its cytotoxic effects, inhibiting HeLa cell proliferation by 42.2% in vitro. In vivo experiments showed that the tumor growth rate of HeLa tumor-bearing mice in the MIP-CTPB group was the slowest, and the tumor volume of the MIP-CTPB treated group was only one-sixth of that of the untreated group.

Immune checkpoints are co-inhibitory ligands that downregulate the activation and function of T cells. They play a key role in tumors by maintaining self-tolerance and modulating immune responses [177]. The reactivation of T-cell immunity by blocking programmed death 1 and PD-L1 immune checkpoints has been extensively applied in cancer therapy [178–180]. However, the N-linked glycosylation of PD-L1 can promote immune evasion by hindering the recognition of polypeptide antigenic regions [181]. To avoid the PD-L1 antibody recognition limit, Zhou et al. presented a new approach for a PD-L1 blockade strategy based on a molecularly imprinted nanostructure called “NanoNiche” to improve T-cell-mediated tumor-killing activity [176]. NanoNiche was prepared by immobilizing PD-L1 N-glycans on gold NPs and then forming a thin imprinting layer of SiO_2_. Moreover, the NanoNiche surface was further functionalized with sialidase to selectively strip SA on the tumor cell membrane, enhancing immune T-cell infiltration. Therefore, it can bind to PD-L1 surface N-glycans, resulting in more efficient PD-L1 blockade and desialylation (Fig. 12C-E). It was confirmed that after NanoNiche treatment, the proliferation of PD-L1-positive MDA-MB-231 human breast cancer cells was significantly inhibited via T-cell killing. In vivo tests revealed favorable therapeutic efficacy in tumor cells with almost no biological toxicity. This technology has the potential to be easily applied in many other immune checkpoint therapies.

HER2, a glycoprotein belonging to the EGFR family, is overexpressed in the outer membrane of several cancer cells [182, 183]. Once bound to growth factors, the extracellular domain of HER2 binds to a second closely related HER family member, forming a heterodimer [62]. The heterodimerization of HER2 enhances the affinity of ligands for other receptors, which ultimately triggers a multistep signaling cascade within the cell that initiates tumorigenesis and metastasis [184]. Thus, preventing the dimerization of HER2 is a valuable method for the effective treatment of cancer. Based on this mechanism, Dong et al. employed MINPs to inhibit the growth of HER2 + breast cancer cells by blocking the HER2 signaling pathway [185]. The HER2-glycan-imprinted nanoMIPs were fabricated by immobilizing HER2 glycans onto FITC-doped SiO_2_ NPs, followed by boronate-affinity-controllable oriented surface imprinting. This process was found to bind nearly all HER2 glycans and suppress the dimerization of HER2 with other HER family members, thereby blocking downstream signaling pathways and inhibiting breast cancer growth. In vitro, the nanoMIPs inhibited HER2 phosphorylation and cancer cell proliferation up to 30%. In vivo, the average tumor volume of the nanoMIP-treated groups was only about half of that of the untreated groups, with no visible biological toxicity (Table 3).

**Table 3:** MIP-based specific recognition intended for cancer therapy

Type of therapy	MIP^a^ components	Template	MIT^a^ strategy	Therapeutic agents	Targeted cancer	Reference
Drug delivery	Monomer: VPBA, AMMH Crosslinker: BAC, EGDMA	SA	Precipitation polymerization (Surface imprinting)	Nitric oxide (NO)	Hepatocellular carcinoma (HepG2 cells)	[129]
	Monomer: NIPAm, TBA, APMA Crosslinker: MBA	C-terminal linear peptide of EFGR	Solid phase method (Surface imprinting)	DOX	Breast cancer (MDA-MA-468 cells)	[126]
	Monomer: TFMA, DMAEMA, NIPA, TBAm Crosslinker: BAC	N-terminal epitope of CD59 glycoprotein	Free radical polymerization (Surface imprinting)	DOX	Breast cancer (MCF-7 cells)	[131]
	Monomer: Zinc acrylate, acrylamide Crosslinker: EDGMA	HER2, DOX	Free radical polymerization (Epitope imprinting)	DOX	Breast cancer (SK-BR-3 cells)	[27]
	Monomer: Zinc acrylate, VPBA Crosslinker: EDGMA	71–80 peptides of FN14, BLM	Free radical polymerization (Epitope imprinting)	Bleomycin	Pancreatic cancer (BxPC-3 cells)	[138]
Photothermal therapy	Monomer: DA, APBA Crosslinker: DA	SA	Self-polymerization (Surface imprinting)	PDA	Breast cancer (MCF-7 cells) Hepatocellular carcinoma (HepG2 cells)	[145]
Photothermal therapy + Drug delivery	Monomer: DA Crosslinker: DA	Epitope of EGFR, DOX	Self-polymerization (Epitope imprinting)	PDA + DOX	Breast cancer (MDA-MB-468 cells) Lung cancer A549 cells	[153]
Photothermal therapy + Immunotherapy	Monomer: DA Crosslinker: DA	HSA	Self-polymerization (Surface imprinting)	PDA + PD-L1 antibody	Breast cancer (4T1 cells)	[157]
Photodynamic therapy	Monomer: PSMAB + CB	SA	Precipitation polymerization (Surface imprinting)	Photosensitizer (CB)	Prostate cancer (DU 145 cells)	[164]
	Monomer: EVAL Crosslinker: BAC	Peptide sequence from PD-L1	Precipitation polymerization (Epitope imprinting)	Photosensitizer (MC540)	Liver cancer (HepG2 cells)	[163]
Photodynamic therapy + Drug delivery	Monomer: NIPAm, TBAm, AAM Crosslinker: BIS	Epitope peptides of CD59, DOX	Free radical polymerization (Epitope imprinting)	Photosensitizer (Ce6) + DOX	Breast cancer (MCF-7 cells)	[162]
Enzyme inhibitor	Monomer: NIPAAm Crosslinker: MBAAm	Trypsin	Solid phase method (Surface imprinting)	MIP_trypsin itself as therapeutic	Trypsin-induced cell injury	[173]
	Monomer: Mercapto-propyl-trimethoxy-silane, N‐(2‐aminoethyl)‐3‐aminopropyltrimethoxy silane	Active center of DHFR	Sol–gel method (Surface imprinting)	MIP-CTPB itself as therapeutic	DHFR-induced DNA synthesis	[175]
Immune checkpoint blockade therapy	Monomer: TEOS	N-glycans of PD-L1 glycoprotein	Surface imprinting	NanoNiche itself as therapeutic	HER2^+^ breast cancer	[176]
Blocking signaling pathway	Monomer: TEOS	N-glycans of HER2 glycoprotein	Surface imprinting	NanoMIP itself as therapeutic	Breast cancer (MDA-MB-231 cells)	[185]

^a^Molecular imprinting technology

## Conclusion

This review covers the most recent advancements in the application of MIPs in cancer diagnostics and therapy. MIP-based technologies are low cost and time efficient, and can be tailored through pre- and post-polymerization modifications. Considering the complexity of most biological fluids, MIPs have repeatedly proven their potential to overcome some of the most challenging aspects in the fabrication of biosensors, such as the stability of the detection probes and the reaction time of the sensing mechanism, with great sensitivity and selectivity. When it comes to the theragnostic applications of MIPs, the capacity of these artificial antibodies to specifically target the moiety of cancer cells and simultaneously allow for the controlled release of chemotherapeutics demonstrates great potential in cancer eradication. Furthermore, as MINPs demonstrate better photostability and higher biocompatibility, their application in PDT and PTT has promising results in reducing the side effects of cancer therapy.

Despite their potential advantages, the use of MIPs in cancer theragnostic is still in its infancy, and there are several reasons for this. One of the main challenges in using MIPs for cancer theragnostic is the complexity and heterogeneity of cancer cell subtypes and tissues. Based on the original and specific subtype, cancer cells and tissues can have a wide range of surface antigens, proteins, and other biomolecules that are potential targets for MIPs, making it difficult to design MIPs that can selectively recognize and bind these targets in a complex biological environment, which limits the successful development of target specific diagnostic system and nanomedicines for cancer treatment. Secondary, the lack of standardization in the synthesis and characterization of MIPs presents a significant challenge, requiring careful optimization of several parameters such as the choice of monomers, template molecules, and cross-linkers. Third, as there is still no standardized method for evaluating the selectivity and binding affinity of MIPs, it remains challenging to compare diagnostic accuracy and affinity among different MIP-based sensors. Lastly but not least, there is still a lack of adequate researches on the prolonged toxicity, biodegradability, compatibility with biological systems, and dispersion of fluids, thus poses challenges to the practical implementation of in vivo applications.

Considering these aspects, though MIPs have shown great potential for cancer theragnostics, there are still several challenges that need to be addressed before they can be widely utilized in clinical practice. For this purpose, further research and development efforts are needed to optimize the synthesis and characterization of MIPs, as well as to validate their efficacy and safety in preclinical and clinical studies. The exceptional characteristics of MIP technology make it a promising tool in cancer diagnostics and therapeutics. In the future, MIP application in sensing, isolating, or targeting will require more real sample testing and in vivo experiments, which will involve the development of new ligands and improved polymer coating techniques. Nonetheless, we believe the application of these artificial antibodies, MIP, in cancer diagnostics and therapeutics is expected to increase.

## Data Availability

Not applicable.

## Abbreviations

MIPs: Molecularly imprinted polymers
ROS: Reactive oxygen species
APS: Ammonium persulfate
TEMED: Tetra-methylethylenediamine
NIPAAm: N-Isopropylacrylamide
MBAAm: N,N′-Methylenebisacrylamide
MINPs: Molecularly imprinted nanoparticles
IDA: Iminodiacetic acid
3-AAPBA: 3-(Acrylamido)phenylboronic acid
B2M: Human β_2_-microglobulin
APTES: Aminopropyltriethoxysilane
TEOS: Tetraethyl orthosilicate
DNA: Deoxyribonucleic acid
RNA: Ribonucleic acid
GCE: Glass carbon electrode
RhoB: Rhodamine B
MAA: Methacrylic acid
AgNPs: Silver nanoparticles
DPV: Differential pulse voltammetry
dsDNA: Double stranded DNA
MPTMS: Mercapto-propyl-trimethoxy-silane
QDs: Quantum dots
MG: Malachite green
RTP: Room temperature phosphorescence
HER2: Human epidermal growth factor receptor 2
LOD: Limit of detection
CEA: Carcinomaembryonic antigen
PDA: Polydopamine
AuNPs: Gold nanoparticles
SMIP: Surface molecularly imprinted polymer
EB: Ethynylbenzene
DA: Dopamine
HRP: Horseradish peroxidase
PSA: Prostate-specific antigen
CA-125: Cancer antigen 125
NPs: Nanoparticles
CDs: Carbon nanodots
EGDMA: Ethylene glycol dimethacrylate
SA: Sialic acid
DOX: Doxorubicin
EGFR: Epidermal growth factor receptors
BS-NPs: Biodegradable silica nanoparticles
MTS: Magnetic-field stimuli-sensitive seed
AC: Alternate current
MTX: Methotrexate
NSMIP: NIR-light responsive surface MIP
FZIF-8/DOX: Fluorescent zeolitic imidazolate framework-8 loaded with doxorubicin
SiNPs: Silicon nanoparticles
PTT: Photothermal therapy
NIR: Near infrared
UCPs: Upconversion nanoparticles
HSA: Human serum albumin
SPARC: Secreted protein acidic and rich in cysteine
PDT: Photodynamic therapy
PS: Photosensitizer
MC540: Merocyanine 540
EVAL: Poly(ethylene-co-vinyl alcohol)
PD-L1: Programmed death-ligand 1
FSiO_2_: Fluorescent silica nanoparticles
Ce6: Chlorin e6
PSMA: Poly(styrene-co-maleic anhydride)
MIP_trypsin: Trypsin-imprinted nanoparticles
DHFR: Dihydrofolate reductase
CTPB: (3-Propanecarboxyl)triphenylphosphonium bromide

## References

[1] Siegel RL, Miller KD, Jemal A (2019). Cancer statistics, 2019. CA Cancer J Clin..

[2] Labi V, Erlacher M (2015). How cell death shapes cancer. Cell Death Dis.

[3] Chen X, Gole J, Gore A, He Q, Lu M, Min J, Yuan Z, Yang X, Jiang Y, Zhang T, Suo C (2020). Non-invasive early detection of cancer four years before conventional diagnosis using a blood test. Nat Commun.

[4] Fiala C, Diamandis EP (2018). Utility of circulating tumor DNA in cancer diagnostics with emphasis on early detection. BMC Med.

[5] Jeong Y, Na K (2018). Synthesis of a gadolinium based-macrocyclic MRI contrast agent for effective cancer diagnosis. Biomat Res.

[6] De Rubis G, Krishnan SR, Bebawy M (2019). Liquid biopsies in cancer diagnosis, monitoring, and prognosis. Trends Pharmacol Sci.

[7] Brown NA, Elenitoba-Johnson KS (2020). Enabling precision oncology through precision diagnostics. Annu Rev Pathol.

[8] Shanmugasundaram KB, Li J, Sina AI, Wuethrich A, Trau M (2022). Toward precision oncology: SERS microfluidic systems for multiplex biomarker analysis in liquid biopsy. Mat Adv.

[9] Lino C, Barrias S, Chaves R, Adega F, Martins-Lopes P, Fernandes JR. Biosensors as diagnostic tools in clinical applications. Biochim Biophys Acta Rev Cancer. 2022;1877:188726. 10.1016/j.bbcan.2022.188726 35367530

[10] Lee JU, Kim WH, Lee HS, Park KH, Sim SJ (2019). Quantitative and specific detection of exosomal miRNAs for accurate diagnosis of breast cancer using a surface-enhanced Raman scattering sensor based on plasmonic head-flocked gold nanopillars. Small.

[11] Jayanthi VS, Das AB, Saxena U (2017). Recent advances in biosensor development for the detection of cancer biomarkers. Biosens Bioelectron.

[12] Haupt K, Medina Rangel PX, Bui BT (2020). Molecularly imprinted polymers: antibody mimics for bioimaging and therapy. Chem Rev.

[13] Ozcelikay G, Kaya SI, Ozkan E, Cetinkaya A, Nemutlu EM, Kır S, Ozkan SA (2022). Sensor-based MIP technologies for targeted metabolomics analysis. TrAC Trend Anal Chem.

[14] Liu Y, Li J, Ou H, Qi D, Hu B, Xu Y, Hu J, Xiong Y, Xia L, Huang JH, Hu X (2022). Identification of new aptamer BC-3 targeting RPS7 from rapid screening for bladder carcinoma. Genes Dis.

[15] Wu L, Wang Y, Xu X, Liu Y, Lin B, Zhang M, Zhang J, Wan S, Yang C, Tan W (2021). Aptamer-based detection of circulating targets for precision medicine. Chem Rev.

[16] Kim DH, Seo JM, Shin KJ, Yang SG (2021). Design and clinical developments of aptamer-drug conjugates for targeted cancer therapy. Biomat Res.

[17] Lowdon JW, Diliën H, Singla P, Peeters M, Cleij TJ, van Grinsven B, Eersels K (2020). MIPs for commercial application in low-cost sensors and assays–An overview of the current status quo. Sens Actuators B Chem.

[18] Mahmoudpour M, Torbati M, Mousavi MM, de la Guardia M, Dolatabadi JE (2020). Nanomaterial-based molecularly imprinted polymers for pesticides detection: Recent trends and future prospects. TrAC Trend Anal Chem.

[19] Dong Q, Yang M, Wang Y, Guan Y, Zhang W, Zhang Y (2023). Peptide-crosslinked molecularly imprinted polymers for efficient separation of immunoglobulin G from human serum. Biomat Sci.

[20] Wang P, Liu J, Ma Y, Tian X, Li Y, Niu X, Luo J, Pan J (2022). Sequential assembly enabled surface precise imprinting on Janus nanosheets for highly specific separation of adenosine 5′-monophosphate. Chem Eng J.

[21] Sun Y, Luo Y, Sun L, Wang XR, Chen LW, Zhang N, Wang Y, Dong LY, Guo H, Wang XH (2023). Improving performance of cell imprinted PDMS by integrating boronate affinity and local post-imprinting modification for selective capture of circulating tumor cells from cancer patients. Biosens Bioelectron.

[22] Matsumoto H, Sunayama H, Kitayama Y, Takano E, Takeuchi T (2019). Site-specific post-imprinting modification of molecularly imprinted polymer nanocavities with a modifiable functional monomer for prostate cancer biomarker recognition. Sci Technol Adv Mater.

[23] Smolinska-Kempisty K, Guerreiro A, Canfarotta F, Cáceres C, Whitcombe MJ, Piletsky S (2016). A comparison of the performance of molecularly imprinted polymer nanoparticles for small molecule targets and antibodies in the ELISA format. Sci Rep.

[24] Wang W, Wang X, Cheng N, Luo Y, Lin Y, Xu W, Du D (2020). Recent advances in nanomaterials-based electrochemical (bio) sensors for pesticides detection. TrAC Trend Anal Chem.

[25] Tuwahatu CA, Yeung CC, Lam YW, Roy VA (2018). The molecularly imprinted polymer essentials: curation of anticancer, ophthalmic, and projected gene therapy drug delivery systems. J Control Release.

[26] Gu Z, Dong Y, Xu S, Wang L, Liu Z (2021). Molecularly imprinted polymer-based smart prodrug delivery system for specific targeting, prolonged retention, and tumor microenvironment-triggered release. Angew Chem.

[27] Wang HY, Cao PP, He ZY, He XW, Li WY, Li YH, Zhang YK (2019). Targeted imaging and targeted therapy of breast cancer cells via fluorescent double template-imprinted polymer coated silicon nanoparticles by an epitope approach. Nanoscale.

[28] Piletsky S, Canfarotta F, Poma A, Bossi AM, Piletsky S (2020). Molecularly imprinted polymers for cell recognition. Trends Biotechnol.

[29] Ali MM, Zhu S, Amin FR, Hussain D, Du Z, Hu L (2022). Molecular imprinting of glycoproteins: From preparation to cancer theranostics. Theranostics.

[30] Yin D, Li X, Ma Y, Liu Z (2017). Targeted cancer imaging and photothermal therapy via monosaccharide-imprinted gold nanorods. Chem Commun.

[31] Zbyradowski M, Duda M, Wisniewska-Becker A, Rajwa W, Fiedor J, Cvetkovic D, Pilch M, Fiedor L (2022). Triplet-driven chemical reactivity of β-carotene and its biological implications. Nat Commun.

[32] Khorsandi K, Hosseinzadeh R, Esfahani H, Zandsalimi K, Shahidi FK, Abrahamse H (2022). Accelerating skin regeneration and wound healing by controlled ROS from photodynamic treatment. Inflamm Regen.

[33] Xu S, Wang L, Liu Z (2021). Molecularly imprinted polymer nanoparticles: an emerging versatile platform for cancer therapy. Angew Chem Int Ed.

[34] Yan H, Row KH (2006). Characteristic and synthetic approach of molecularly imprinted polymer. Int J Mol Sci.

[35] Hasanah AN, Safitri N, Zulfa A, Neli N, Rahayu D (2021). Factors Affecting preparation of molecularly imprinted polymer and methods on finding template-monomer interaction as the key of selective properties of the materials. Molecules.

[36] Chen H, Guo J, Wang Y, Dong W, Zhao Y, Sun L (2022). Bio-Inspired Imprinting Materials for Biomedical Applications. Adv Sci.

[37] Saylan Y, Yilmaz F, Özgür E, Derazshamshir A, Yavuz H, Denizli A (2017). Molecular imprinting of macromolecules for sensor applications. Sensors (Basel).

[38] Arabi M, Ostovan A, Li J, Wang X, Zhang Z, Choo J, Chen L (2021). Molecular imprinting: green perspectives and strategies. Adv Mater.

[39] Ertürk G, Mattiasson B (2017). Molecular imprinting techniques used for the preparation of biosensors. Sensors (Basel).

[40] Reville EK, Sylvester EH, Benware SJ, Negi SS, Berda EB (2022). Customizable molecular recognition: advancements in design, synthesis, and application of molecularly imprinted polymers. Polym Chem.

[41] Takeuchi T, Haginaka J (1999). Separation and sensing based on molecular recognition using molecularly imprinted polymers. J Chromatogr B Biomed Sci Appl.

[42] Schirhagl R (2014). Bioapplications for molecularly imprinted polymers. Anal Chem.

[43] Vasapollo G, Sole RD, Mergola L, Lazzoi MR, Scardino A, Scorrano S, Mele G (2011). Molecularly imprinted polymers: present and future prospective. Int J Mol Sci.

[44] Parlak O, Keene ST, Marais A, Curto VF, Salleo A (2018). Molecularly selective nanoporous membrane-based wearable organic electrochemical device for noninvasive cortisol sensing. Sci Adv..

[45] Chen L, Wang X, Lu W, Wu X, Li J (2016). Molecular imprinting: perspectives and applications. Chem Soc Rev.

[46] Sajini T, Mathew B (2021). A brief overview of molecularly imprinted polymers: Highlighting computational design, nano and photo-responsive imprinting. Talanta Open.

[47] Nahhas AF, Webster TJ (2021). The promising use of nano-molecular imprinted templates for improved SARS-CoV-2 detection, drug delivery and research. J Nanobiotechnology.

[48] Yang K, Li S, Liu L, Chen Y, Zhou W, Pei J, Liang Z, Zhang L, Zhang Y (2019). Epitope imprinting technology: progress, applications, and perspectives toward artificial antibodies. Adv Mater.

[49] Sorribes-Soriano A, Esteve-Turrillas FA, Armenta S, Amorós P, Herrero-Martínez JM (2019). Amphetamine-type stimulants analysis in oral fluid based on molecularly imprinting extraction. Anal Chim Acta.

[50] Yang LL, Li YJ, Jiang ZF, Li QY, Ma RR, He JY, Zhou LD, Zhang QH, Yuan CS (2021). Investigating two distinct dummy templates molecularly imprinted polymers as paclitaxel adsorbent in synthesis system and releaser in biological samples. Microchem J.

[51] Zhou Z, Kong D, Zhu H, Wang N, Wang Z, Wang Q, Liu W, Li Q, Zhang W, Ren Z (2018). Preparation and adsorption characteristics of an ion-imprinted polymer for fast removal of Ni (II) ions from aqueous solution. J Hazard Mater.

[52] Banan K, Ghorbani-Bidkorbeh F, Afsharara H, Hatamabadi D, Landi B, Keçili R, Sellergren B (2022). Nano-sized magnetic core-shell and bulk molecularly imprinted polymers for selective extraction of amiodarone from human plasma. Anal Chim Acta.

[53] Xu X, Chen X, Yang L, Zhao Y, Zhang X, Shen R, Sun D, Qian J (2020). Film-like bacterial cellulose based molecularly imprinted materials for highly efficient recognition and adsorption of cresol isomers. Chem Eng J.

[54] Smolinska-Kempisty K, Guerreiro A, Czulak J, Piletsky S (2019). Negative selection of MIPs to create high specificity ligands for glycated haemoglobin. Sens Actuators B Chem.

[55] Ҫimen D, Bereli N, Günaydın S, Denizli A (2022). Molecular imprinted nanoparticle assisted surface plasmon resonance biosensors for detection of thrombin. Talanta.

[56] Lv P, Xie D, Zhang Z (2018). Magnetic carbon dots based molecularly imprinted polymers for fluorescent detection of bovine hemoglobin. Talanta.

[57] Ghani NT, Abdulla H, Rizk MS, Dena AS, El Nashar RM (2019). Molecularly imprinted polymer/reduced graphene oxide-based carbon-paste sensor for highly sensitive determination of the anti-HCV drug daclatasvir dihydrochloride. Sens Actuators B Chem.

[58] Ma Y, Yin Y, Ni L, Miao H, Wang Y, Pan C, Tian X, Pan J, You T, Li B, Pan G (2021). Thermo-responsive imprinted hydrogel with switchable sialic acid recognition for selective cancer cell isolation from blood. Bioact Mater.

[59] Zangiabadi M, Zhao Y (2020). Selective binding of complex glycans and glycoproteins in water by molecularly imprinted nanoparticles. Nano Lett.

[60] Garnier M, Sabbah M, Ménager C, Griffete N (2021). Hybrid molecularly imprinted polymers: the future of nanomedicine?. Nanomaterials.

[61] Dabrowski M, Lach P, Cieplak M, Kutner W (2018). Nanostructured molecularly imprinted polymers for protein chemosensing. Biosens Bioelectron.

[62] Xu J, Miao H, Wang J, Pan G (2020). Molecularly imprinted synthetic antibodies: from chemical design to biomedical applications. Small.

[63] Gao J, Yan L, Yan Y, Chen L, Lu J, Xing W, Yu C, Chen M, Meng M, Yan Y, Wu Y (2022). Solvent-driven controllable molecularly imprinted membrane with switched selectivity and fast regenerability enabled by customized bifunctional monomers. Chem Eng J.

[64] Li D, Tu T, Yang M, Xu C (2018). Efficient preparation of surface imprinted magnetic nanoparticles using poly (2-anilinoethanol) as imprinting coating for the selective recognition of glycoprotein. Talanta.

[65] Wang P, Zhu H, Liu J, Ma Y, Yao J, Dai X, Pan J (2019). Double affinity integrated MIPs nanoparticles for specific separation of glycoproteins: A combination of synergistic multiple bindings and imprinting effect. Chem Eng J.

[66] Wang X, Yu S, Liu W, Fu L, Wang Y, Li J, Chen L (2018). Molecular imprinting based hybrid ratiometric fluorescence sensor for the visual determination of bovine hemoglobin. ACS Sens.

[67] Kidakova A, Boroznjak R, Reut J, Öpik A, Saarma M, Syritski V (2020). Molecularly imprinted polymer-based SAW sensor for label-free detection of cerebral dopamine neurotrophic factor protein. Sens Actuators B Chem.

[68] Yang JC, Cho CH, Choi DY, Park JP, Park J (2022). Microcontact surface imprinting of affinity peptide for electrochemical impedimetric detection of neutrophil gelatinase-associated lipocalin. Sens Actuators B Chem.

[69] Silva D, de Sousa HC, Gil MH, Santos LF, Amaral RA, Saraiva JA, Salema-Oom M, Alvarez-Lorenzo C, Serro AP, Saramago B (2021). Imprinted hydrogels with LbL coating for dual drug release from soft contact lenses materials. Mater Sci Eng C.

[70] Kalecki J, Iskierko Z, Cieplak M, Sharma PS (2020). Oriented immobilization of protein templates: a new trend in surface imprinting. ACS Sens.

[71] Liu L, Dong C, Li X, Li S, Ma B, Zhao B, Li X, Liang Z, Yang K, Zhang L, Zhang Y (2020). Antibody-free hydrogel with the synergistic effect of cell imprinting and Boronate affinity: toward the selective capture and release of undamaged circulating tumor cells. Small.

[72] Sarpong KA, Xu W, Huang W, Yang W (2019). The development of molecularly imprinted polymers in the clean-up of water pollutants: a review. Am J Anal Chem.

[73] Whitcombe MJ, Chianella I, Larcombe L, Piletsky SA, Noble J, Porter R, Horgan A (2011). The rational development of molecularly imprinted polymer-based sensors for protein detection. Chem Soc Rev.

[74] Dong C, Shi H, Han Y, Yang Y, Wang R, Men J (2021). Molecularly imprinted polymers by the surface imprinting technique. Eur Polym J.

[75] Gao FX, Ma XT, He XW, Li W-Y, Zhang YK (2013). Smart surface imprinting polymer nanospheres for selective recognition and separation of glycoprotein. Colloids Surf A Physicochem Eng Asp.

[76] Sanadgol N, Wackerlig J (2020). Developments of smart drug-delivery systems based on magnetic molecularly imprinted polymers for targeted cancer therapy: a short review. Pharmaceutics.

[77] Xing R, Ma Y, Wang Y, Wen Y, Liu Z (2019). Specific recognition of proteins and peptides via controllable oriented surface imprinting of boronate affinity-anchored epitopes. Chem Sci.

[78] Dar KK, Shao S, Tan T, Lv Y (2020). Molecularly imprinted polymers for the selective recognition of microorganisms. Biotechnol Adv.

[79] Teixeira SP, Reis RL, Peppas NA, Gomes ME, Domingues RM (2021). Epitope-imprinted polymers: Design principles of synthetic binding partners for natural biomacromolecules. Sci Adv..

[80] Guo Z, Xing R, Zhao M, Li Y, Lu H, Liu Z (2021). Controllable engineering and functionalizing of nanoparticles for targeting specific proteins towards biomedical applications. Adv Sci.

[81] Ge Y, Butler B, Mirza F, Habib-Ullah S, Fei D (2013). Smart molecularly imprinted polymers: recent developments and applications. Macromol Rapid Commun.

[82] Xu S, Lu H, Zheng X, Chen L (2013). Stimuli-responsive molecularly imprinted polymers: versatile functional materials. J Mater Chem C.

[83] Ding X, Heiden PA (2014). Recent developments in molecularly imprinted nanoparticles by surface imprinting techniques. Macromol Mater Eng.

[84] Caldara M, Lowdon JW, Rogosic R, Arreguin-Campos R, Jimenez-Monroy KL, Heidt B, Tschulik K, Cleij TJ, Diliën H, Eersels K, van Grinsven B (2021). Thermal detection of glucose in urine using a molecularly imprinted polymer as a recognition element. ACS Sens.

[85] Liu R, Poma A (2021). Advances in molecularly imprinted polymers as drug delivery systems. Molecules.

[86] Yu W, Hurley J, Roberts D, Chakrabortty SK, Enderle D, Noerholm M, Breakefield XO, Skog JK (2021). Exosome-based liquid biopsies in cancer: opportunities and challenges. Ann Oncol.

[87] Marrugo-Ramírez J, Mir M, Samitier J (2018). Blood-based cancer biomarkers in liquid biopsy: a promising non-invasive alternative to tissue biopsy. Int J Mol Sci.

[88] Tan WC, Nerurkar SN, Cai HY, Ng HH, Wu D, Wee YT, Lim JC, Yeong J, Lim TK (2020). Overview of multiplex immunohistochemistry/immunofluorescence techniques in the era of cancer immunotherapy. Cancer Commun.

[89] Rolfo C, Mack PC, Scagliotti GV, Baas P, Barlesi F, Bivona TG, Herbst RS, Mok TS, Peled N, Pirker R, Raez LE (2018). Liquid biopsy for advanced non-small cell lung cancer (NSCLC): a statement paper from the IASLC. J Thorac Oncol.

[90] Heitzer E, Haque IS, Roberts CE, Speicher MR (2019). Current and future perspectives of liquid biopsies in genomics-driven oncology. Nat Rev Genet.

[91] Wu J, Hu S, Zhang L, Xin J, Sun C, Wang L, Ding K, Wang B (2020). Tumor circulome in the liquid biopsies for cancer diagnosis and prognosis. Theranostics.

[92] Seo JH, Lee JW, Cho D (2018). The market trend analysis and prospects of cancer molecular diagnostics kits. Biomater Res.

[93] Lu H, Xu S, Guo Z, Zhao M, Liu Z (2021). Redox-responsive molecularly imprinted nanoparticles for targeted intracellular delivery of protein toward cancer therapy. ACS Nano.

[94] Liao Z, Peng J, Chen S, Zhang P, Chen H, Feng D, Zhang T, Ye K, Deng Y, Dong Y, Geng L (2022). Sensitive fluorescent sensor for the fuzzy exosomes in serum based on the exosome imprinted polymer sandwiched with aggregation induced emission. Sens Actuators B Chem.

[95] Feng D, Ren M, Miao Y, Liao Z, Zhang T, Chen S, Ye K, Zhang P, Ma X, Ni J, Hu X (2022). Dual selective sensor for exosomes in serum using magnetic imprinted polymer isolation sandwiched with aptamer/graphene oxide based FRET fluorescent ignition. Biosens Bioelectron.

[96] Bhakta S, Mishra P (2021). Molecularly imprinted polymer-based sensors for cancer biomarker detection. Sens Actuators Rep.

[97] Lai Y, Deng Y, Yang G, Li S, Zhang C, Liu X (2018). Molecular imprinting polymers electrochemical sensor based on AuNPs/PTh modified GCE for highly sensitive detection of carcinomaembryonic antigen. J Biomed Nanotechnol.

[98] Mo G, Qin D, Jiang X, Zheng X, Mo W, Deng B (2020). A sensitive electrochemiluminescence biosensor based on metal-organic framework and imprinted polymer for squamous cell carcinoma antigen detection. Sens Actuators B Chem.

[99] Bach DH, Lee SK, Sood AK (2019). Circular RNAs in cancer. Mol Ther Nucleic Acids.

[100] Salehi M, Sharifi M (2018). Exosomal miRNAs as novel cancer biomarkers: challenges and opportunities. J Cell Physiol.

[101] Hashemi-Moghaddam H, Kashi M, Mowla SJ, Nouraee N (2016). Separation of microRNA 21 as a cancer marker from glioblastoma cell line using molecularly imprinted polymer coated on silica nanoparticles. J Sep Sci.

[102] Arslan T, Güney O (2020). Ratiometric sensor based on imprinted quantum dots-cationic dye nanohybrids for selective sensing of dsDNA. Anal Biochem.

[103] You M, Yang S, Tang W, Zhang F, He P (2018). Molecularly imprinted polymers-based electrochemical DNA biosensor for the determination of BRCA-1 amplified by SiO2@ Ag. Biosens Bioelectron.

[104] Lahcen AA, Rauf S, Aljedaibi A, de Oliveira Filho JI, Beduk T, Mani V, Alshareef HN, Salama KN (2021). Laser-scribed graphene sensor based on gold nanostructures and molecularly imprinted polymers: application for Her-2 cancer biomarker detection. Sens Actuators B Chem.

[105] Lin X, Wang Y, Wang L, Lu Y, Li J, Lu D, Zhou T, Huang Z, Huang J, Huang H, Qiu S (2019). Interference-free and high precision biosensor based on surface enhanced Raman spectroscopy integrated with surface molecularly imprinted polymer technology for tumor biomarker detection in human blood. Biosens Bioelectron.

[106] Zhou L, Wang Y, Xing R, Chen J, Liu J, Li W, Liu Z (2019). Orthogonal dual molecularly imprinted polymer-based plasmonic immunosandwich assay: a double characteristic recognition strategy for specific detection of glycoproteins. Biosens Bioelectron.

[107] Wang Y, Kan X (2021). Sensitive and selective “signal-off” electrochemiluminescence sensing of prostate-specific antigen based on an aptamer and molecularly imprinted polymer. Analyst.

[108] Karami P, Bagheri H, Johari-Ahar M, Khoshsafar H, Arduini F, Afkhami A (2019). Dual-modality impedimetric immunosensor for early detection of prostate-specific antigen and myoglobin markers based on antibody-molecularly imprinted polymer. Talanta.

[109] Rebelo TS, Costa R, Brandão AT, Silva AF, Sales MG, Pereira CM (2019). Molecularly imprinted polymer SPE sensor for analysis of CA-125 on serum. Anal Chim Acta.

[110] Zhu Y, An Y, Li R, Zhang F, Wang Q, He P (2020). Double imprinting-based electrochemical detection of mimetic exosomes. J Electroanal Chem.

[111] Liu L, Liu J, Zhou W, Sui Z, Liu J, Yang K, Zhang L, Liang Z, Zhang Y (2022). An artificial antibody for exosome capture by dull template imprinting technology. J Mater Chem B.

[112] Mori K, Hirase M, Morishige T, Takano E, Sunayama H, Kitayama Y, Inubushi S, Sasaki R, Yashiro M, Takeuchi T (2019). A pretreatment-free, polymer-based platform prepared by molecular imprinting and post-imprinting modifications for sensing intact exosomes. Angew Chem.

[113] Takeuchi T, Mori K, Sunayama H, Takano E, Kitayama Y, Shimizu T, Hirose Y, Inubushi S, Sasaki R, Tanino H (2020). Antibody-conjugated signaling nanocavities fabricated by dynamic molding for detecting cancers using small extracellular vesicle markers from tears. J Am Chem Soc.

[114] Demir B, Lemberger MM, Panagiotopoulou M, Medina Rangel PX, Timur S, Hirsch T, Tse Sum Bui B, Wegener J, Haupt K (2018). Tracking hyaluronan: molecularly imprinted polymer coated carbon dots for cancer cell targeting and imaging. ACS Appl Mater Interfaces.

[115] Beyer S, Kimani M, Zhang Y, Verhassel A, Sternbæk L, Wang T, Persson JL, Härkönen P, Johansson E, Caraballo R, Elofsson M (2022). Fluorescent molecularly imprinted polymer layers against sialic acid on silica-coated polystyrene cores—assessment of the binding behavior to cancer cells. Cancers.

[116] King HA, El-Sharif HF, Matia-González AM, Iadevaia V, Fowotade A, Reddy SM, Gerber AP (2017). Generation of ribosome imprinted polymers for sensitive detection of translational responses. Sci Rep.

[117] Siegel RL, Miller KD, Fuchs HE, Jemal A (2022). Cancer statistics, 2022. CA Cancer J Clin.

[118] Yu MK, Park J, Jon S (2012). Targeting strategies for multifunctional nanoparticles in cancer imaging and therapy. Theranostics.

[119] Kemp JA, Kwon YJ (2021). Cancer nanotechnology: current status and perspectives. Nano Converg.

[120] Li T, Shi W, Yao J, Hu J, Sun Q, Meng J, Wan J, Song H, Wang H (2022). Combinatorial nanococktails via self-assembling lipid prodrugs for synergistically overcoming drug resistance and effective cancer therapy. Biomater Res.

[121] Parisi OI, Ruffo M, Malivindi R, Vattimo AF, Pezzi V, Puoci F (2020). Molecularly imprinted polymers (MIPs) as theranostic systems for sunitinib controlled release and self-monitoring in cancer therapy. Pharmaceutics.

[122] Sun T, Zhang YS, Pang B, Hyun DC, Yang M, Xia Y (2021). Engineered nanoparticles for drug delivery in cancer therapy. Angew Chem Int Ed Engl.

[123] Chabner BA, Roberts TG (2005). Chemotherapy and the war on cancer. Nat Rev Cancer.

[124] Zhong L, Li Y, Xiong L, Wang W, Wu M, Yuan T, Yang W, Tian C, Miao Z, Wang T, Yang S (2021). Small molecules in targeted cancer therapy: advances, challenges, and future perspectives. Signal Transduct Target Ther.

[125] Zhang H (2020). Molecularly imprinted nanoparticles for biomedical applications. Adv Mater.

[126] Canfarotta F, Lezina L, Guerreiro A, Czulak J, Petukhov A, Daks A, Smolinska-Kempisty K, Poma A, Piletsky S, Barlev NA (2018). Specific drug delivery to cancer cells with double-imprinted nanoparticles against epidermal growth factor receptor. Nano Lett.

[127] Shi J, Kantoff PW, Wooster R, Farokhzad OC (2017). Cancer nanomedicine: progress, challenges and opportunities. Nat Rev Cancer.

[128] Rosenblum D, Joshi N, Tao W, Karp JM, Peer D (2018). Progress and challenges towards targeted delivery of cancer therapeutics. Nat Commun.

[129] Liu T, Qiao Z, Wang J, Zhang P, Zhang Z, Guo DS, Yang X (2019). Molecular imprinted S-nitrosothiols nanoparticles for nitric oxide control release as cancer target chemotherapy. Colloids Surf B Biointerfaces.

[130] Matsumoto A, Cabral H, Sato N, Kataoka K, Miyahara Y (2010). Assessment of tumor metastasis by the direct determination of cell-membrane sialic acid expression. Angew Chem Int Ed.

[131] Qin YT, Feng YS, Ma YJ, He XW, Li WY, Zhang YK (2020). Tumor-sensitive biodegradable nanoparticles of molecularly imprinted polymer-stabilized fluorescent zeolitic imidazolate framework-8 for targeted imaging and drug delivery. ACS Appl Mater Interfaces.

[132] Zhang M, Hu W, Cai C, Wu Y, Li J, Dong S (2022). Advanced application of stimuli-responsive drug delivery system for inflammatory arthritis treatment. Mater Today Bio.

[133] Liu LT, Chen MJ, Yang HL, Huang ZJ, Tang Q, Chow CF, Gong CB, Zu MH, Xiao B (2020). An NIR-light-responsive surface molecularly imprinted polymer for photoregulated drug release in aqueous solution through porcine tissue. Mater Sci Eng C.

[134] Zhang Y, Wang Q, Zhao X, Ma Y, Zhang H, Pan G (2023). Molecularly imprinted nanomaterials with stimuli responsiveness for applications in biomedicine. Molecules.

[135] Kubo T, Tachibana K, Naito T, Mukai S, Akiyoshi K, Balachandran J, Otsuka K (2018). Magnetic field stimuli-sensitive drug release using a magnetic thermal seed coated with thermal-responsive molecularly imprinted polymer. ACS Biomater Sci Eng.

[136] Li L, Chen L, Zhang H, Yang Y, Liu X, Chen Y (2016). Temperature and magnetism bi-responsive molecularly imprinted polymers: Preparation, adsorption mechanism and properties as drug delivery system for sustained release of 5-fluorouracil. Mater Sci Eng C Mater Biol Appl.

[137] Patra JK, Das G, Fraceto LF, Campos EV, Rodriguez-Torres MD, Acosta-Torres LS, Diaz-Torres LA, Grillo R, Swamy MK, Sharma S, Habtemariam S (2018). Nano based drug delivery systems: recent developments and future prospects. J Nanobiotechnology.

[138] Jia C, Zhang M, Zhang Y, Ma ZB, Xiao NN, He XW, Li WY, Zhang YK (2019). Preparation of dual-template epitope imprinted polymers for targeted fluorescence imaging and targeted drug delivery to pancreatic cancer BxPC-3 cells. ACS Appl Mater Interfaces.

[139] Liu Y, Bhattarai P, Dai Z, Chen X (2019). Photothermal therapy and photoacoustic imaging via nanotheranostics in fighting cancer. Chem Soc Rev.

[140] Hao S, Han H, Yang Z, Chen M, Jiang Y, Lu G, Dong L, Wen H, Li H, Liu J, Wu L (2022). Recent advancements on photothermal conversion and antibacterial applications over MXenes-based materials. Nano-Micro Lett.

[141] Dai Y, Sun Z, Zhao H, Qi D, Li X, Gao D, Li M, Fan Q, Shen Q, Huang W (2021). NIR-II fluorescence imaging guided tumor-specific NIR-II photothermal therapy enhanced by starvation mediated thermal sensitization strategy. Biomaterials.

[142] Zhang SY, Zhou ZR, Qian RC (2021). Recent progress and perspectives on cell surface modification. Chem Asian J.

[143] Zhang L, Oudeng G, Wen F, Liao G (2022). Recent advances in near-infrared-II hollow nanoplatforms for photothermal-based cancer treatment. Biomater Res.

[144] Chen J, Ning C, Zhou Z, Yu P, Zhu Y, Tan G, Mao C (2019). Nanomaterials as photothermal therapeutic agents. Prog Mater Sci.

[145] Wang S, Ding Y, Wang H, Li W, Xu W, Sun P, Huang W, Chen Y, Gu J, Lin P, Ma L (2022). Molecularly imprinted upconversion nanoparticles for active tumor targeting and microinvasive photothermal therapy. J Mater Sci.

[146] Zhang H, Zhang M, Zhang X, Gao Y, Ma Y, Chen H, Wan J, Li C, Wang F, Sun X (2022). Enhanced postoperative cancer therapy by iron-based hydrogels. Biomater Res.

[147] Wang J, Sui L, Huang J, Miao L, Nie Y, Wang K, Yang Z, Huang Q, Gong X, Nan Y, Ai K (2021). MoS2-based nanocomposites for cancer diagnosis and therapy. Bioact Mater.

[148] Fan W, Yung B, Huang P, Chen X (2017). Nanotechnology for multimodal synergistic cancer therapy. Chem Rev.

[149] Tian H, Zhang T, Qin S, Huang Z, Zhou L, Shi J, Nice EC, Xie N, Huang C, Shen Z (2022). Enhancing the therapeutic efficacy of nanoparticles for cancer treatment using versatile targeted strategies. J Hematol Oncol.

[150] Xu Y, Hu X, Guan P, Du C, Tian Y, Ding S, Li Z, Yan C (2019). A novel controllable molecularly imprinted drug delivery system based on the photothermal effect of graphene oxide quantum dots. J Mater Sci.

[151] Poinard B, Neo SZ, Yeo EL, Heng HP, Neoh KG, Kah JC (2018). Polydopamine nanoparticles enhance drug release for combined photodynamic and photothermal therapy. ACS Appl Mater Interfaces.

[152] Ostovan A, Arabi M, Wang Y, Li J, Li B, Wang X, Chen L (2022). Greenificated molecularly imprinted materials for advanced applications. Adv Mater.

[153] Liu H, Deng Z, Bu J, Zhang Y, Zhang Z, He Y, Li T, Gao P, Yang Y, Zhong S (2021). Capsule-like molecular imprinted polymer nanoparticles for targeted and chemophotothermal synergistic cancer therapy. Colloids Surf B Biointerfaces.

[154] Yue J, Mei Q, Wang P, Miao P, Dong WF, Li L (2022). Light-triggered multifunctional nanoplatform for efficient cancer photo-immunotherapy. J Nanobiotechnology.

[155] Li Z, Lai X, Fu S, Ren L, Cai H, Zhang H, Gu Z, Ma X, Luo K (2022). Immunogenic cell death activates the tumor immune microenvironment to boost the immunotherapy efficiency. Adv Sci.

[156] Huang X, Lu Y, Guo M, Du S, Han N (2021). Recent strategies for nano-based PTT combined with immunotherapy: from a biomaterial point of view. Theranostics.

[157] Ma J, Zhang Y, Sun H, Ding P, Chen D (2022). Fabrication of human serum albumin–imprinted photothermal nanoparticles for enhanced immunotherapy. J Mater Chem B.

[158] Dougherty TJ, Gomer CJ, Henderson BW, Jori G, Kessel D, Korbelik M, Moan J, Peng Q (1998). Photodynamic therapy. JNCI.

[159] Park IK, Ju DB, Babu A, Lee JC, Pyung YJ, Cho CS, Kim HJ (2022). In vitro photodynamic therapy of methylene blue-loaded acetyl resistant starch nanoparticles. Biomater Res.

[160] Liu Q, Wu LZ (2017). Recent advances in visible-light-driven organic reactions. Natl Sci Rev.

[161] Ai X, Mu J, Xing B (2016). Recent advances of light-mediated theranostics. Theranostics.

[162] Peng H, Qin YT, He XW, Li WY, Zhang YK (2020). Epitope molecularly imprinted polymer nanoparticles for chemo-/photodynamic synergistic cancer therapy guided by targeted fluorescence imaging. ACS Appl Mater Interfaces.

[163] Lin CC, Lin HY, Thomas JL, Yu JX, Lin CY, Chang YH, Lee MH, Wang TL (2021). Embedded upconversion nanoparticles in magnetic molecularly imprinted polymers for photodynamic therapy of hepatocellular carcinoma. Biomedicines.

[164] Peng R, Luo Y, Cui Q, Yao C, Xu W, Li L (2022). Conjugated oligomer-directed formation of hollow nanoparticles for targeted photokilling cancer cells under hypoxia. Adv Opt Mater.

[165] Zhang C, Qin WJ, Bai XF, Zhang XZ (2020). Nanomaterials to relieve tumor hypoxia for enhanced photodynamic therapy. Nano Today.

[166] Jing X, Yang F, Shao C, Wei K, Xie M, Shen H, Shu Y (2019). Role of hypoxia in cancer therapy by regulating the tumor microenvironment. Mol Cancer.

[167] Rajalakshmi PS, Alvi SB, Begum N, Veeresh B, Rengan AK (2021). Self-assembled fluorosome–polydopamine complex for efficient tumor targeting and commingled photodynamic/photothermal therapy of triple-negative breast cancer. Biomacromol.

[168] Zhang H, Jiang J, Zhang H, Zhang Y, Sun P (2013). Efficient synthesis of molecularly imprinted polymers with enzyme inhibition potency by the controlled surface imprinting approach. ACS Macro Lett.

[169] Cutivet A, Schembri C, Kovensky J, Haupt K (2009). Molecularly imprinted microgels as enzyme inhibitors. J Am Chem Soc.

[170] Guerreiro A, Poma A, Karim K, Moczko E, Takarada J, de Vargas-Sansalvador IP, Turner N, Piletska E, de Magalhães CS, Glazova N, Serkova A (2014). Influence of Surface-Imprinted Nanoparticles on Trypsin Activity. Adv Healthc Mater.

[171] Zheng C, Zhang XL, Liu W, Liu B, Yang HH, Lin ZA, Chen GN (2013). A selective artificial enzyme inhibitor based on nanoparticle-enzyme interactions and molecular imprinting. Adv Mater.

[172] Yamamoto H, Iku S, Adachi Y, Imsumran A, Taniguchi H, Nosho K, Min Y, Horiuchi S, Yoshida M, Itoh F, Imai K (2003). Association of trypsin expression with tumour progression and matrilysin expression in human colorectal cancer. J Pathol.

[173] Xu J, Miao H, Zou L, Tse Sum Bui B, Haupt K, Pan G (2021). Evolution of molecularly imprinted enzyme inhibitors: from simple activity inhibition to pathological cell regulation. Angew Chem.

[174] Hopper AT, Brockman A, Wise A, Gould J, Barks J, Radke JB, Sibley LD, Zou Y, Thomas S (2019). Discovery of selective Toxoplasma gondii dihydrofolate reductase inhibitors for the treatment of toxoplasmosis. J Med Chem.

[175] Qin YT, Ma YJ, Feng YS, He XW, Li WY, Zhang YK (2021). Targeted mitochondrial fluorescence imaging-guided tumor Antimetabolic therapy with the imprinted polymer nanomedicine capable of specifically recognizing Dihydrofolate reductase. ACS Appl Mater Interfaces.

[176] Zhou ZR, Wang XY, Jiang L, Li DW, Qian RC (2021). Sialidase-conjugated “NanoNiche” for efficient immune checkpoint blockade therapy. ACS Appl Bio Mater.

[177] Alissafi T, Hatzioannou A, Legaki AI, Varveri A, Verginis P (2019). Balancing cancer immunotherapy and immune-related adverse events: the emerging role of regulatory T cells. J Autoimmun.

[178] Woods DM, Sodré AL, Villagra A, Sarnaik A, Sotomayor EM, Weber J (2015). HDAC Inhibition Upregulates PD-1 Ligands in Melanoma and Augments Immunotherapy with PD-1 BlockadeHDAC Inhibition Upregulates PD-1 Ligands in Melanoma. Cancer Immunol Res.

[179] Akinleye A, Rasool Z (2019). Immune checkpoint inhibitors of PD-L1 as cancer therapeutics. J Hematol Oncol.

[180] Lee NK, Kim SN, Park CG (2021). Immune cell targeting nanoparticles: a review. Biomater Res.

[181] Wang YN, Lee HH, Hsu JL, Yu D, Hung MC (2020). The impact of PD-L1 N-linked glycosylation on cancer therapy and clinical diagnosis. J Biomed Science.

[182] Oh DY, Bang YJ (2020). HER2-targeted therapies—a role beyond breast cancer. Nat Rev Clin Oncol.

[183] Meric-Bernstam F, Johnson AM, Dumbrava EE, Raghav K, Balaji K, Bhatt M, Murthy RK, Rodon J, Piha-Paul SA (2019). Advances in HER2-Targeted Therapy: Novel Agents and Opportunities Beyond Breast and Gastric CancerAdvances in HER2-Targeted Therapy. Clin Cancer Res.

[184] Dhritlahre RK, Saneja A (2021). Recent advances in HER2-targeted delivery for cancer therapy. Drug Discov Today.

[185] Dong Y, Li W, Gu Z, Xing R, Ma Y, Zhang Q, Liu Z (2019). Inhibition of HER2-positive breast cancer growth by blocking the HER2 signaling pathway with HER2-glycan-imprinted nanoparticles. Angew Chem Int Ed.

